# The σ1 Receptor and the HINT1 Protein Control α2δ1 Binding to Glutamate NMDA Receptors: Implications in Neuropathic Pain

**DOI:** 10.3390/biom11111681

**Published:** 2021-11-12

**Authors:** María Rodríguez-Muñoz, Elsa Cortés-Montero, Yara Onetti, Pilar Sánchez-Blázquez, Javier Garzón-Niño

**Affiliations:** 1 Neuropharmacology, Department of Translational Neuroscience, Cajal Institute, CSIC, 28002 Madrid, Spain; mrodriguez@cajal.csic.es (M.R.-M.); elsa.cortes@cajal.csic.es (E.C.-M.); yara.onetti@ub.edu (Y.O.); psb@cajal.csic.es (P.S.-B.); 2 Instituto Cajal, Consejo Superior de Investigaciones Científicas (CSIC), Doctor Arce 37, 28002 Madrid, Spain

**Keywords:** mechanical allodynia, sigma receptor type 1, HINT1 protein

## Abstract

Nerve injury produces neuropathic pain through the binding of α2δ1 proteins to glutamate *N*-methyl-D-aspartate receptors (NMDARs). Notably, mice with a targeted deletion of the sigma 1 receptor (*σ1R)* gene do not develop neuropathy, whereas mice lacking the histidine triad nucleotide-binding protein 1 (*Hint1)* gene exhibit exacerbated allodynia. σ1R antagonists more effectively diminish neuropathic pain of spinal origin when administered by intracerebroventricular injection than systemically. Thus, in mice subjected to unilateral sciatic nerve chronic constriction injury (CCI), we studied the participation of σ1Rs and HINT1 proteins in the formation of α2δ1-NMDAR complexes within the supraspinal periaqueductal gray (PAG). We found that δ1 peptides required σ1Rs in order to interact with the NMDAR NR1 variant that contains the cytosolic C1 segment. σ1R antagonists or low calcium levels provoke the dissociation of σ1R-NR1 C1 dimers, while they barely affect the integrity of δ1-σ1R-NR1 C1 trimers. However, HINT1 does remove δ1 peptides from the trimer, thereby facilitating the subsequent dissociation of σ1Rs from NMDARs. In σ1R^−/−^ mice, CCI does not promote the formation of NMDAR-α2δ1 complexes and allodynia does not develop. The levels of α2δ1-σ1R-NMDAR complexes increase in HINT1^−/−^ mice and after inducing CCI, degradation of α2δ1 proteins is observed. Notably, σ1R antagonists but not gabapentinoids alleviate neuropathic pain in these mice. During severe neuropathy, the metabolism of α2δ1 proteins may account for the failure of many patients to respond to gabapentinoids. Therefore, σ1Rs promote and HINT1 proteins hinder the formation α2δ1-NMDAR complexes in the PAG, and hence, the appearance of mechanical allodynia depends on the interplay between these proteins.

## 1. Introduction

Persistent anomalous activation of glutamate *N*-methyl-D-aspartate receptors (NMDARs) typically accompanies different types of neuropathic pain, as characterized by tactile allodynia and hyperalgesia [1]. NMDARs are under allosteric regulation of different endogenous and exogenous molecules, of which the alpha2delta1 (α2δ1) protein plays a decisive role in the neuropathy promoted by the over activation of NMDARs [2]. Proteins of the α2δ family, α2δ1, α2δ2, α2δ3, and α2δ4 are derived from distinct genes (*Cacna2d1-4*) with different sequences. These *α2δ* genes encode a single precursor protein, which is post-translationally processed into two proteins in the endoplasmic reticulum (ER): a larger N terminal α2 protein that is essentially extracellular; and the smaller δ peptide in the carboxyl region that contains a transmembrane domain and an intracellular region [3,4]. Finally, the α2 and δ proteins are re-assembled via disulfide bonds [5]. The α2 protein is heavily glycosylated [3,6], and enzymatic deglycosylation of the reduced α2δ complex produces peptides of 100 and 17 kDa.

Murine α2δ1 is a 1067 residue protein of about 115 kDa found at high levels in the anterior cingulate cortex, amygdala, and periaqueductal gray (PAG), and at lower levels in the spinal cord (SC) [7]. As a consequence of nerve damage, α2δ1 proteins and α2δ1-NMDAR complexes augment considerably in the dorsal root ganglia (DRG) and SC, giving rise to neuropathic pain [8]. Gabapentinoids, such as gabapentin and pregabalin, are widely used to alleviate the symptoms of neuropathic pain and epilepsy [9,10,11]. These drugs bind to the α2 region of the α2δ1 and α2δ2 variants, but not to α2δ3 [12]. While, experimental α2δ1 overexpression potentiates NMDAR activity in spinal dorsal horn neurons, provoking pain hypersensitivity, disruption of the *α2δ1* gene prevents nerve injury from enhancing NMDAR activity, suggesting that when coupled to NMDARs these α2δ1 proteins are the therapeutic target of gabapentinoids [8].

Notably, α2δ1 proteins and the type 1 sigma receptors (σ1Rs) physically interact with NMDARs to promote calcium permeation and ultimately, neuropathic pain [8,13]. Accordingly, σ1R antagonists alleviate neuropathic allodynia and inflammatory hyperalgesia in animal models of pain that involve NMDAR activation [14,15,16]. Similarly, σ1R^−/−^ mice do not develop allodynia in different paradigms of neuropathic pain, such as sciatic nerve chronic constriction injury (CCI) [17], paclitaxel induced pain [18], SC contusion injury [19], or spare nerve injury [20]. Mice lacking the histidine triad nucleotide-binding protein 1 (*Hint1)* gene display altered NMDAR activity [21] and they are more susceptible to CCI-induced mechanical hypersensitivity than their wild-type (WT) littermates. Moreover, HINT1 regulators can alleviate CCI-induced mechanical allodynia for several days in WT mice [22]. Hence, σ1Rs appear to promote and HINT1 proteins dampen NMDAR-mediated neuropathic pain. Both the σ1R and HINT1 protein are widely expressed in nervous tissue, detected at high levels in areas that are associated with pain control [23]. Furthermore, both these regulatory proteins bind to the NR1 subunit of the NMDAR that carries the C1 domain within the cytosolic C0-C1-C2(2′) tail [24]. This domain coordinates the activity of NMDARs with that of G-protein coupled receptors (GPCRs), such as the mu-opioid receptor (MOR) or cannabinoid type 1 receptor (CB1R) [25].

The relationship between α2δ1 proteins and NMDARs in nerve injury has been characterized extensively in the DRG and SC [8]. Nevertheless, drugs regulating σ1R or HINT1 activity efficiently alleviate neuropathic pain when administered by the intracerebroventricular (icv) route [22,25]. In fact, neuropathic pain persists even after spinal ascending nociceptive signals remit, suggesting a role for supraspinal neural structures in this syndrome. The periaqueductal gray (PAG) matter is a midbrain structure strongly implicated in the nociceptive and emotional aspects of pain processing. Specifically, the ventrolateral PAG controls upstream spinal nociceptive signals, regulating their strength in the midbrain and the dorsal SC (substantia gelatinosa) through inhibitory descending pathways [26]. This control may be impaired by spinal nerve injury, which causes upstream changes in PAG glutamatergic neurotransmission, with an upregulation of NMDARs and hypofunction of α-amino-3-hydroxy-5-methyl-4-isoxazolepropionic receptors (AMPARs). These alterations reduce PAG descending pain inhibition and consequently, they prolong the duration of neuropathic pain [27]. Thus, we have studied the role of σ1R and HINT1 proteins in the formation of α2δ1-NMDAR complexes in this brain structure promoted by nerve injury.

## 2. Materials and Methods

### 2.1. Animals and Drugs

Two strains of mice were used in these studies, CD1 and 129. Wild type male albino CD1 mice served as controls for the homozygous CD1 male sigma 1 receptor (σ1R^−/−^) knockout mice; as the σ1R^−/−^ mice were backcrossed (N10 generation) onto a CD1 albino genetic background (ENVIGO, Milano, Italy). Wild type male 129 mice served as controls for their homozygous, male, 129 HINT1 protein (HINT1^−/−^) knockout mice littermates. HINT1^−/−^ mice on a 129 mouse genetic background were generously supplied by I.B.Weinstein/J.B.Wang and bred at our animal facility. The genotypes of the WT and KO mice were confirmed by PCR. The mice used in these experiments were produced from heterozygous breeding pairs and assigned randomly to the different experiments. All mouse housing, breeding, and experimental protocols were in strict accordance to the European Community guidelines for the Care and Use of Laboratory Animals (Council Directive 2010/63/EU) and Spanish law (RD53/2013) regulating animal research. The use of drugs, the experimental design, and the sample size determination was approved by the CSIC Ethical Committee for Research (PROEX 317/16). The mice were maintained at 22 °C on a diurnal 12 h light/dark cycle, and provided free access to food and water. To reduce the risk of social stress, mice from the same litter were grouped together and remained in these groups throughout the study. The mice were also provided extra space for comfort, as well as nesting material (e.g., soft paper and cardboard refuge) and small pieces of chewable wood. The mice were used when they were between 6 and 10 weeks of age, and the number of animals used in this study were: CD1 wild type 180, CD1 σ1R^−/−^ 40, 129 wild type 38, 129 HINT1^−/−^ 56.

The compounds used in this study were: S1RA (#16279, Cayman Chemical, Ann Arbor, MI, USA), gabapentin (#0806, Tocris Bioscience, Bristol, UK), pregabalin (#3775, Tocris Bioscience, Bristol, UK), BD1047 (#0956, Tocris Bioscience, Bristol, UK), memantine (#0773, Tocris Bioscience, Bristol, UK), pregnenolone sulfate (#P162, Sigma, Madrid, Spain), PRE084 (#0589, Tocris Bioscience, Bristol, UK). The drugs were dissolved in saline and the doses and treatment intervals were selected based on previous studies and pilot assays. To facilitate selective and straightforward access to their targets, the compounds were injected (4 µL) into the lateral ventricles of mice as described previously [28]. Animals were lightly anesthetized and injections were performed with a 10 µL Hamilton syringe at a depth of 3 mm at a point of 2 mm lateral and 2 mm caudal to the bregma. The compounds were infused at a rate of 1 µL every 5 s, after which the needle was maintained in place for an additional 10 s.

### 2.2. Chronic Constriction Injury (CCI)

After testing mice for their basal mechanical sensitivity, CCI was performed under isoflurane/oxygen anesthesia [22] using a modified version of the Bennett and Xie procedure [29]. Briefly, a 0.5-cm incision was made in the right midthigh, the biceps femoris muscle was separated and the sciatic nerve was exposed proximal to its trifurcation. Two ligatures (5/0 braided silk suture, #70014: Lorca Marin, Murcia, Spain) were tied around this nerve approximately 1 mm apart until a short flick of the ipsilateral hind limb was observed. The incision was then closed in layers with a 4–0 Ethicon silk suture. The tactile pain thresholds of both the ipsilateral and contralateral hind paws were then assessed at different time intervals post-surgery. The mice were placed individually in a transparent plastic cage with a wire mesh bottom that allowed access to their paws. After a habituation period of 20 min, a mechanical stimulus was delivered to the plantar surface from below the floor of the test chamber to measure allodynia using an automatic von Frey apparatus (#37450: Ugo Basile, Comerio, Italy). A steel rod (0.5 mm diameter) was pushed against the hind paw over a 10 s period, increasing the force from 0 to 10 g. When the mouse withdrew its hind paw, the mechanical stimulus was automatically stopped and the force at which withdrawal occurred was recorded. Animals were sacrificed at the time allodynia peaked, which was observed seven days after surgery.

### 2.3. Immunoprecipitation and Western Blotting

The preparation of the membranes and the immunoprecipitation assays were performed as described previously [30,31]. The specificity and efficacy of the antibodies used in immunoprecipitation assays have been addressed elsewhere [32,33]. Briefly, the brain and SC structures were collected and homogenized in 10 volumes of 25 mM Tris-HCl [pH 7.5] and 0.32 M sucrose supplemented with a 0.2 mM phenylmethylsulphonyl fluoride (PMSF). The homogenate was centrifuged at 1000× *g* for 10 min to remove the nuclear fraction. The supernatant (S1) was centrifuged twice at 20,000× *g* for 20 min to obtain the crude synaptosomal pellet (P2). The final pellet was diluted in Tris buffer supplemented with a 0.2 mM PMSF and a protease inhibitor cocktail (#P8340, Sigma, St. Louis, MO, USA), then divided into aliquots and processed for protein determinations.

For immunoprecipitation studies, about 800 µg of protein from the P2 pellet was solubilized by sonication at 4 °C (two cycles of 10 s each) in a 2 mL volume containing 50 mM Tris-HCl [pH 7.5], 50 mM NaCl, 1% Nonidet P-40, phosphatase inhibitor mixture (#P2850, Sigma, St. Louis, MO, USA), and a protease inhibitor cocktail (#P8340, Sigma, St. Louis, MO, USA). Solubilization was continued overnight at 4 °C and the lysates were then cleared with streptavidin agarose (#17-5113-01, GE Healthcare, Chicago, IL, USA) for 1 h at 4 °C. The solubilized proteins were then incubated overnight at 4 °C with affinity-purified biotinylated IgGs raised against NR1, NR2A, and NR2B subunits of the NMDAR. The samples were incubated with streptavidin agarose for 2 h and then centrifuged for 5 min at 4300× *g*. The agarose pellets recovered were subjected to five cycles of washing and resuspension in Nonidet P-40 buffer, followed by centrifugation. To detach the immunocomplexes, the samples were heated with 2× Laemmli buffer (#1610737, Bio-Rad, Madrid, Spain) with added reducing agents, for 10 min at 100 °C. The mixture was cooled to room temperature and the streptavidin agarose was separated in a centrifugal filter with a pore size of 0.22 µm (Ultrafree-MC #UFC30GV0S: Merck-Millipore, Madrid, Spain). The immunoprecipitated proteins were recovered and resolved by SDS-PAGE on 10 cm × 10 cm × 1.5 mm gel slabs (12% total acrylamide concentration, 2.6% bisacrylamide cross-linker), and the separated proteins were then transferred onto 0.2 µm polyvinylidene difluoride (PVDF) membranes (#162-0176, Bio-Rad, Madrid, Spain). The membranes were probed overnight at 6 °C with the selected primary antibodies diluted in Tris-buffered saline [pH 7.6; TBS] + 0.05% Tween 20 (TTBS), detecting antibody binding with secondary antibodies conjugated to horseradish peroxidase.

The images of the Western blots and the antibody binding were visualized by chemiluminescence (#170-5061, Bio-Rad, Madrid, Spain) and recorded on an ImageQuantTM LAS 500 apparatus (GE Healthcare, Chicago, IL, USA) typically selecting the area containing the target protein in each blot. The software automatically calculates the optimal exposure time for each of the areas specified to provide the strongest possible signal for accurate quantification of the sample. Protein immunosignals were measured using the area of the strongest signal for each group of samples studied (average optical density of the pixels within the object area/mm^2^; AlphaEase FC software), the grey values of the means were then normalized within the 8 bit/256 grey levels [(256-computed value)/computed value]. Equal loading was verified and when necessary adjusted to α-tubulin. In the immunoprecipitation studies, the secondary antibodies were directed to either the heavy or light IgG chains of the primary antibodies, as needed and thus, the secondary antibodies reacted primarily with the separated IgG heavy or light chains of the accompanying antibodies used for immunoprecipitation providing a control for the gel loading of the samples [30].

The antibodies used for immunoprecipitation were directed against amino acid sequences in the extracellular domains of the membrane receptors and labeled with biotin following the manufacturer’s instructions (#21217; ThermoScientific, Waltham, MA, USA): affinity purified IgGs against the NMDAR NR1 subunit (483–496: KFGTQERVNNSNKK; GenScript Co., Piscataway, NJ, USA), the NMDAR NR2A subunit (343–356: WDGKDLSFTEEGYQ, GenScript Co., Piscataway, NJ, USA), and NMDAR NR2B subunit (19–32: AVSGSKARSQKSAP, GenScript Co., Piscataway, NJ, USA). The primary antibodies used in Western blotting were raised against: NMDAR NR1 (#MAB1586, Merck-Millipore, Burlington, MA, USA); NMDAR NR1 C1 (#AB5046, Merck-Millipore, Burlington, MA, USA); NMDAR NR2A (#AB1555P, Merck-Millipore, Burlington, MA, USA); NR2B (#MA1-2014, ThermoScientific, Waltham, MA, USA); α2(δ1) Nt (#C5105, Sigma Aldrich, St. Louis, MO, USA); α2(δ1) inner sequence (#SAB2107922, Sigma Aldrich, St. Louis, MO, USA); (α2)δ1 (#HPA008621, Sigma Aldrich, St. Louis, MO, USA); α2(δ2) (#A10267, Abclonal, Woburn, MA, USA); (α2)δ2 (#HPA071829, Sigma Aldrich, St. Louis, MO, USA), α-tubulin (#ab7291, Abcam, Cambridge, UK).

### 2.4. PNGase F Digestion of Immunoprecipitated Proteins

The NR2A and NR2B subunits were immunoprecipitated from the solubilized P2 fraction of the PAG as described above. The agarose pellets underwent five cycles of washing, followed by centrifugation and resuspension in 1 mL of Nonidet P-40 buffer. At the end of this process, immune complexes were resuspended and solubilized in 100 mM NaH_2_PO_4_ [pH 7.7], 1 mM EDTA, 1% β-mercaptoethanol, 0.5% SDS and 1 mM dithiothreitol, and heated at 100 °C for 10 min. The solubilized material was supplemented with 0.65% octylthioglucoside to help remove any SDS from the proteins and then incubated for 18 h at 37 °C with PNGase F (5 units/10 μg of protein, #V4831: Promega, Madrid, Spain). The samples were then concentrated, solubilized in Laemmli buffer, separated on a 10% SDS-polyacrylamide gel, and the α2(δ1) and α2(δ2) immunosignals were probed in Western blots.

### 2.5. Recombinant Protein Expression

The coding region of the full-length murine voltage-dependent calcium channel subunit delta1 (α2)δ1 (NM_001110846: residues 3123–3560) and its C terminal truncated variants, of the voltage-dependent calcium channel subunit delta2 (α2)δ2 (AF247139: residues 3456–3911), σ1R (AF004927), HINT1 (NM_008248), and the cytosolic C0-C1-C2 region of the glutamate NMDAR NR1 subunit (NM_008169: residues 834–938), were amplified by RT-PCR using total RNA isolated from the mouse brain as the template. Specific primers containing an upstream Sgf I restriction site and a downstream Pme I restriction site were used, as described previously [24]. The PCR products were cloned downstream of the Glutathione S-transferase (GST)/HaloTag^®^ coding sequence in the Flexi^®^ Vector (Promega, Madison, WI, USA) and the tobacco etch virus protease (TEV) protease site, and the proteins were identical to the GenBank™ sequences when sequenced. The vector was introduced into the E. coli BL21 (KRX #L3002, Promega, Madison, WI, USA) and clones were selected on solid medium containing ampicillin. After a 3 h induction at room temperature (RT) in the presence of 1 mM isopropyl β-D-1-thiogalactopyranoside (IPTG) and 0.1% rhamnose, the cells were collected by centrifugation and maintained at −80 °C. The fusion proteins were purified under native conditions on GStrap FF columns (#17-5130-01, GE Healthcare, Spain) or with HaloLink Resin (#G1915, Promega, Madison, WI, USA). When necessary, the fusion proteins retained were cleaved on the column with ProTEV protease (#V605A, Promega, Madison, WI, USA) and further purification was achieved by high-resolution ion exchange (#780-0001Enrich Q, BioRad, Hercules, CA, USA). Sequences were confirmed by automated capillary sequencing. Recombinant calmodulin (#208694, Merck-Millipore, Burlington, MA, USA) was obtained from commercial sources.

### 2.6. In Vitro Interactions between Recombinant Proteins and the Pull-Down of Recombinant Proteins

The recombinant σ1R (100 nM) was incubated for 30 min at RT with either Sepharose 4B (#17-0120-01, GE Healthcare; negative control) alone or together with the immobilized (α2)δ1 peptides in 300 µL of a buffer containing 50 mM Tris-HCl [pH 7.4] and 0.2% CHAPS in the presence of increasing amounts of CaCl_2_. After incubation, the pellets were recovered by centrifugation, washed three times in the presence of 2.5 mM CaCl_2_, solubilized in 2× Laemmli buffer with added β-mercaptoethanol, and analyzed in Western blots. This protocol was also carried out to assess the competition between HINT1/CaM and higher concentrations of σ1R for (α2)δ1 peptides. Whether the (α2)δ2 peptides interacted with NR1 C1, σ1R, HINT1, or CaM, and the calcium effect on these associations, was also studied using the aforementioned protocol.

The relevance of the (α2)δ1 C terminal sequence in the association with σ1R was addressed by generating truncated (α2)δ1 C terminal sequences (–10 aa or –30 aa). These peptides were incubated with σ1R (30 or 100 nM) in 300 µL of a buffer containing 50 mM Tris-HCl [pH 7.4] and 0.2% CHAPS in the presence of 2.5 mM CaCl_2_, and mixed by rotation for 30 min at RT. This protocol was also carried out to assess whether the (α2)δ1 peptides directly bind to NR1 subunits. NR1 C0-C1-C2 or NR1 C0-C2 C-terminus variants (100 nM) were incubated with (α2)δ1 in the presence of 2.5 mM CaCl_2_. These interactions were carried out in presence of 1% or 10% DMSO, or adding 30 μM of a peptide mapping to the C0 (849–858: QLAFAAVNVW; PepMic Co., Suzhou, China) or C1 region of NR1 subunit (879–888: TFRAITSTLA; PepMic Co., Suzhou, China), which facilitates the binding of CaM to NR1 C1 subunits (24). The influence of the peptides mapping to the C0 or C1 region of NR1 subunit on the association of truncated (α2)δ1 peptides (–30 aa) with NR1 C1 was also evaluated. The purity of all these peptides was higher than 95%.

The role of σ1Rs on the association of NR1 C1 with (α2)δ1 peptides was addressed through preincubation of 100 nM σ1R with agarose-NR1 C1 in 300 μL of a buffer containing 50 mM Tris-HCl [pH 7.4] and 0.2% CHAPS in the presence of 2.5 mM CaCl_2_, and mixed by rotation for 30 min at RT. After removal of the unbound σ1Rs, agarose-attached NR1-σ1R complexes were incubated for a further 30 min at RT with rotation in the presence of 100 nM (α2)δ1 peptides in a reaction volume of 300 μL containing 50 mM Tris-HCl [pH 7.4], 0.2% CHAPS and 2.5 mM CaCl_2_. In a set of assays, 100 nM NR1 C1 was added to agarose-(α2)δ1-σ1R complexes and incubated for 30 min at RT. Agarose-bound proteins were obtained by centrifugation, washed three times, solubilized in 2× Laemmli buffer plus β-mercaptoethanol, and analyzed in Western blots. The implication of HINT1 or CaM in the binding of (α2)δ1 to the NR1 C1 subunits was also studied. To ensure the CaM binding site on the NR1 C0-C1-C2 sequence was available, the assay was performed in presence of 30 μM of a peptide mapping to the C0 region (849–858: QLAFAAVNVW).

The influence of HINT1 on NR1 C1-σ1R-(α2)δ1 trimeric complexes was also studied. The agarose-attached NR1 C1-σ1R-(α2)δ1 complexes were incubated for 30 min at RT with rotation in the presence of 100 nM HINT1 in a reaction volume of 300 μL containing 50 mM Tris-HCl [pH 7.4], 0.2% CHAPS, and 2.5 mM CaCl_2_. The effect of increasing the concentration of the σ1R ligands on the NR1 C1-σ1R dimer and the NR1 C1-σ1R-(α2)δ1 trimer was also evaluated. In another set of assays the effect of σ1R ligands (1 µM) on σ1R-(α2)δ1 interaction was analyzed. The agarose-attached NR1 C1-σ1R or NR1 C1-σ1R-(α2)δ1 complexes were incubated for 30 min at RT with rotation in the presence of increasing concentrations of the drugs in a final reaction volume of 300 μL containing 50 mM Tris-HCl [pH 7.4] and 0.2% CHAPS in the presence of 2.5 mM CaCl_2_. The detached proteins recovered in the aforementioned procedures were resolved by SDS-PAGE in 4–12% Bis-Tris gels (#NP0341, Invitrogen, Fisher Scientific, Hampton, NH, USA), with MES SDS as the running buffer (#NP0002, Invitrogen, Fisher Scientific, Hampton, NH, USA). The proteins were transferred to 0.2 μm PVDF membranes (#162-0176, BioRad, Hercules, CA, USA) that were then probed overnight at 6 °C with primary antibodies diluted in Tris-buffered saline [pH 7.7; TBS] + 0.05% Tween 20 (TTBS): anti-NMDAR NR1 C1 (#AB5046, Merck-Millipore, Madrid, Spain), anti-(α2)δ1 (#HPA008621, Sigma Aldrich, St. Louis, MO, USA), anti-σ1R (#42-3300, Invitrogen, Fisher Scientific, Hampton, NH, USA), anti-HINT1 (aa 93–106; Inmunostep, Salamanca, Spain) and anti-CaM (#05-173, Merck-Millipore, Madrid, Spain). All primary antibodies were detected as described above. Because all the assays were performed with recombinant proteins, the antibodies detected a single band of the expected size, which was used for the subsequent densitometry analysis, see above. Accordingly, no other regions of the blots provided information and were routinely excluded from the analysis.

### 2.7. Statistical Analysis

The signals from the Western blots were expressed as the change relative to the controls, which were assigned an arbitrary value of 1. Statistical analyses were performed using the Sigmaplot/SigmaStat v.14.5 package [statistical package for the social sciences (SPSS) Science Software, Erkrath, Germany] and the level of significance was considered as *p* < 0.05. The data were analyzed using an one-way analysis of variance (ANOVA) followed by the Holm-Sidak multiple comparisons test. Statistical significance (α) was defined as *p* < 0.05. The power (1-β) of the tests performed at α = 0.05 was always > 0.80 (80%).

## 3. Results

When solubilized in Laemmli buffer supplemented with the reducing agent β-mercaptoethanol, mature α2δ proteins resolved as two independent protein subunits on SDS-PAGE, the large α2 N terminal region and the δ peptide. Thus, the name of the α2δ1-2 subunit under study will be accompanied by that of the partner protein indicated in parentheses, i.e., α2(δ1-2) and (α2)δ1-2.

**Figure 1:**
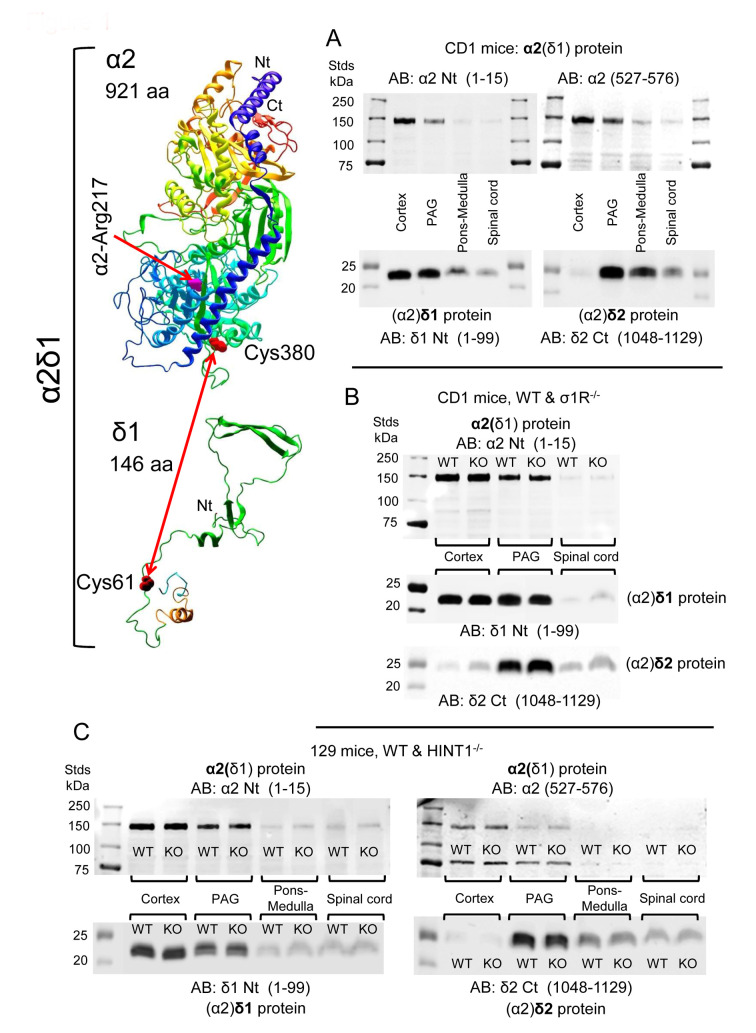
Expression of α2(δ1), (α2)δ1, and (α2)δ2 in the cerebral cortex, PAG, pons-medulla, and SC of CD1 and 129 mice. (**A**) CD1 WT mice (*n* = 5). (**B**) CD1 WT (*n* = 5) and CD1 σ1R^−/−^ mice (*n* = 6). (**C**) 129 WT (n = 6) and 129 HINT1^−/−^ (*n* = 6) mice. Mouse brain and SC structures were collected and P2 fractions enriched in synaptosomes were obtained. About 60 μg protein/lane was resolved by SDS-PAGE and examined in Western blots probed with antibodies against α2(δ) proteins and (α2)δ peptides, as described in the Methods. Further details in Supplemental Figures S1 and S2. The 3D structure of α2(δ1) and (α2)δ1 were generated with NovaFold v. 17 (DNASTAR), in which α2-Arg217 is indicated as a pink tube, and α2-Cys380 and δ1-Cys61 are shown as red tubes.

The distribution of α2(δ1) was studied in the CNS with two antibodies, one directed against the N terminal sequence and the other against the internal region from amino acids (aa) 527–576. The (α2)δ1 peptide was detected with an antibody directed against the initial 1–99 aa, and the (α2)δ2 peptide with an antibody mapping to the C terminal region (1048–1129 aa). Since, multiple bands were evident following direct detection with the antibody against the α2(δ2) subunit, some not of the predicted sizes, these data are not shown. In CD1 mice, the α2(δ1) protein was more strongly expressed in the cortex, followed by the PAG, and with the lowest levels observed in the pons-medulla and SC. An identical pattern of expression was found for the associated (α2)δ1 peptide. The (α2)δ2 peptide was barely detected in the cortex but it was most strongly expressed in the PAG, followed by the pons-medulla and SC (Figure 1A, Supplementary Material Figure S1).

**Figure 2:**
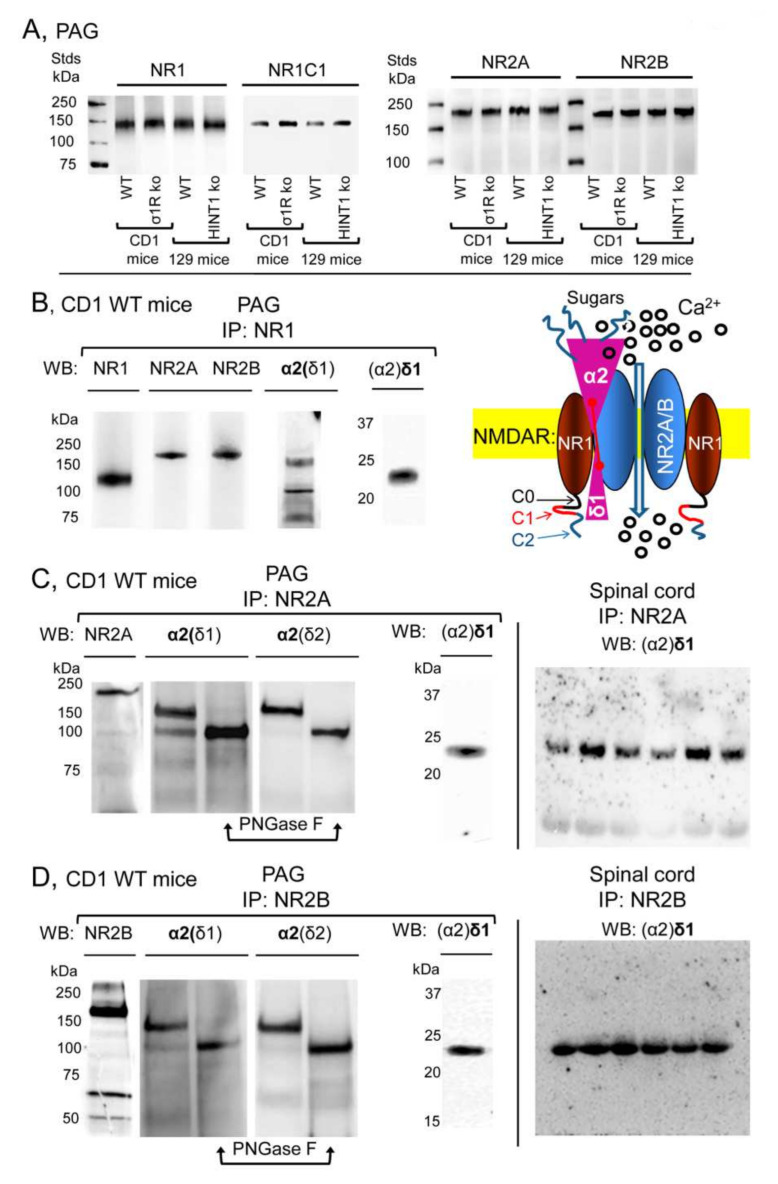
Expression of NR1, NR2A, and NR2B subunits of glutamate NMDARs in mouse PAG: Co-precipitation with α2(δ1), α2(δ2), and (α2)δ1 proteins. (**A**) PAG P2 fractions enriched in synaptosomes from the CD1 WT, CD1 σ1R^−/−^, 129 WT, and 129 HINT1^−/−^ mice of Figure 1B,C, were used. About 60 μg protein/lane was resolved by SDS-PAGE and examined in Western blots that were probed with antibodies against NR1, NR1 C1, NR2A, and NR2B NMDAR subunits, as described in Methods. The assay was repeated twice with comparable outcomes. Immunoprecipitation assays on CD1 WT mice (*n* = 12): PAG and SC membranes from CD1 WT mice were solubilized with 1% NP-40 and incubated overnight at 4 °C with affinity-purified biotinylated IgGs raised against the (**B**) NR1, (**C**) NR2A, or (**D**) NR2B subunits. Protein complexes were immunoprecipitated (IP) with streptavidin agarose, resolved by SDS-PAGE and visualized in Western blots. The expected size of the α2(δ1) and α2(δ2) is approximately 100 kDa; however, the bands detected usually appeared as a doublet of 100–140 kDa. (**C**,**D**), The material associated with the NR2A or NR2B subunits was subjected to deglycosylation with PNGase F, which depleted the α2(δ) 140 kDa band in favor of a 100 kDa band. In SC synaptosomes, (α2)δ1-2 immunosignals were enriched at NR2A/B subunits (each lane was loaded with solubilized spinal cord tissue from a single CD1 WT mouse, for more details see Section 2). Inset: Diagram of the proposed association of α2δ1 proteins with NMDARs. The α2(δ1) protein and the (α2)δ1 peptide are bridged by a disulfide bond, and both bind to the NR1-NR2A/B dimer. The heavily glycosylated α2(δ1) protein remains outside the membrane interacting with external sequences of the NMDAR, while the (α2)δ1 peptide contains a transmembrane region followed by the C terminal region, which interacts with the cytosolic regions of the NR1 C0-C1-C2(2’) subunits. Stimuli like nerve injury promote changes in the NR1 C1 subunit that augment the stability of its association with the (α2)δ1 peptide. Consequently, calcium permeation increases and persists, causing NMDAR over activation and allodynia. Plasma membrane in yellow; NR1 subunits in brown; NR2 subunits in blue; α2δ1 proteins in pink; spheres indicate calcium ions.

Because we studied both CD1 σ1R^−/−^ and 129 HINT1^−/−^ mice, the levels of the α2δ1-2 proteins and NMDAR subunits were also evaluated in these genetically-modified mice and compared to those in their respective CD1 and 129 WT controls. Accordingly, CNS levels of the α2δ1-2 protein were similar in CD1 mice lacking *σ1R* gene and their control CD1 WT mice (Figure 1B, Supplementary Material Figure S1), and the targeted deletion of the *HINT1* gene also failed to significantly alter the expression of the α2δ1–2 proteins in 129 mice (Figure 1C, Supplementary Material Figure S2). The levels of NMDAR NR1 and NR2A subunits were comparable in the PAG of CD1 WT mice and 129 WT mice, and deletion of the *σ1R* or *HINT1* genes did not alter the expression of these proteins. There were stronger signals for the NR1 variant carrying the cytosolic C1 segment in CD1 σ1R^−/−^ and 129 HINT1^−/−^ mice than in their WT controls. The signal corresponding to the NR2B subunits increased in the PAG of 129 HINT1^−/−^ mice relative to that in 129 WT mice (Figure 2A). These observations are consistent with previous reports [13,21,25], and their statistical significance was verified here through analysis of immunoprecipitation assays.

In the PAG of CD1 naïve mice, the NR1 NMDAR subunit co-precipitated with the NR2A, NR2B, α2(δ1), and (α2)δ1 proteins (Figure 2B). The NR2A and NR2B subunits also co-precipitated with the α2(δ1) and (α2)δ1 proteins (Figure 2C,D). In reducing SDS-PAGE, the mobility of the α2(δ1) subunit indicated a size of about 150 kDa, larger than the predicted 107 kDa based on its aa sequence. Previous studies indicate that functional α2(δ1) is heavily glycosylated [3,6] and indeed, after exposing the co-precipitated α2(δ1) to PNGase F its apparent size diminished to 100 kDa (Figure 2C,D, Supplementary Material Figure S3). Although, direct detection of (α2)δ1 produced only weak signals in the SC, its immunoreactivity augmented strongly when it was co-precipitated with NR2A/B subunits (Figure 2C,D). The diagram suggests that α2δ1 interacts with the NR1 and NR2A/B subunits, but with stronger affinity to the latter, and that the C terminal region of (α2)δ1 is decisive to stabilize this interaction [8].

**Figure 3:**
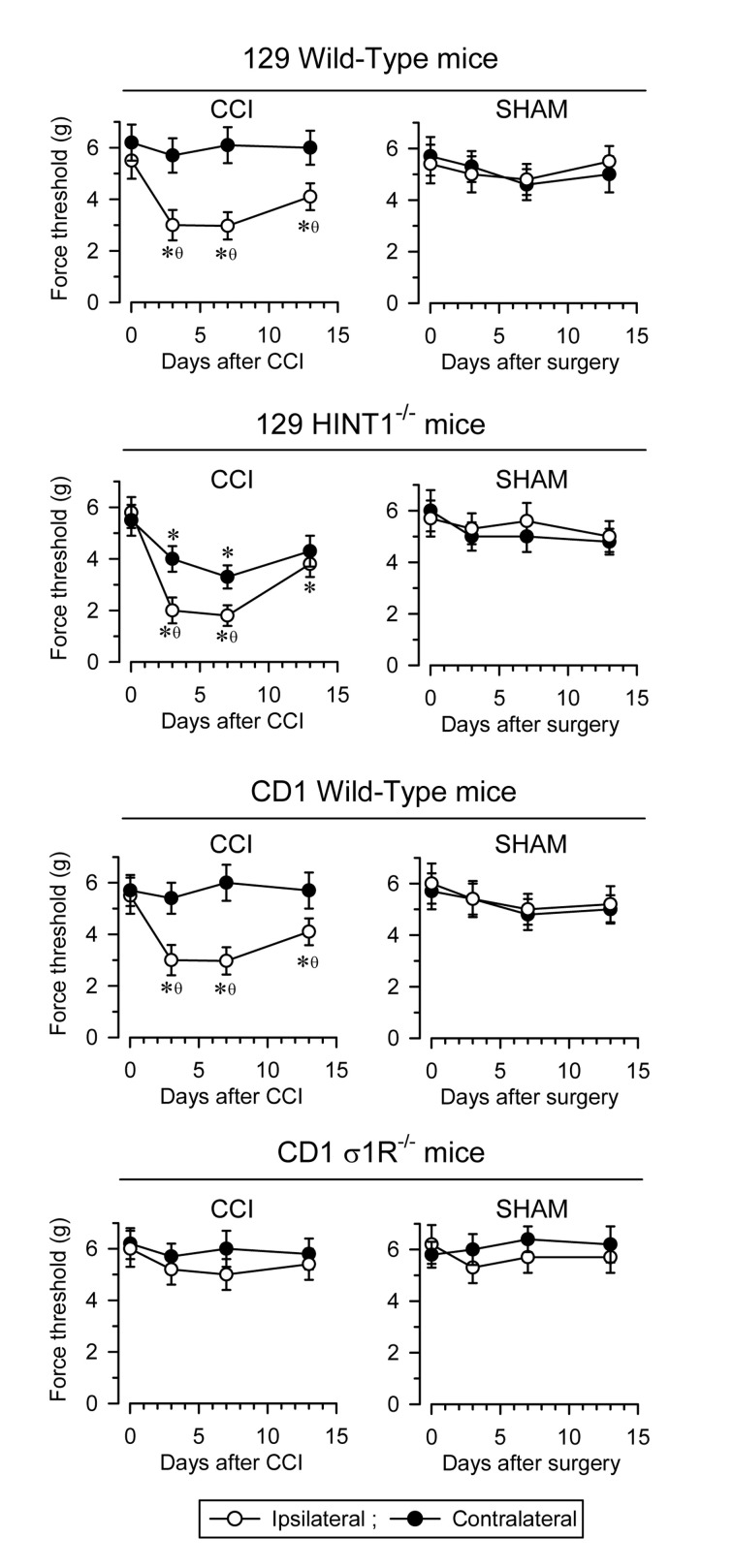
Induction of mechanical allodynia in 129 WT, 129 HINT1^−/−^, CD1 WT, and CD1 σ1R^−/−^ mice. Chronic constriction injury (CCI) of the sciatic nerve causes neuropathic pain in mice. For each mouse strain, sham (*n* = 6) and CCI (*n* = 6) operated mice were examined. The paw withdrawal thresholds after CCI, corresponding to contralateral and ipsilateral paws were measured before (indicated as 0) and 3, 7, and 14 days after surgery. The force (in grams) at which the mice withdrew their paws in response to von Frey hair stimulation was determined as an index of mechanical allodynia. The data are shown as the mean ± SD of six mice: * significantly different at the corresponding time interval relative to the nociceptive threshold on day 0; θ indicates a significant difference relative to the contralateral paw. All the data were analyzed by pairwise Holm-Sidak multiple comparison tests following ANOVA: *p*  <  0.05, 1 − β > 0.80.

In rodents, unilateral sciatic nerve CCI is an accepted model to study neuropathic pain. In CD1 and 129 WT mice, pain develops over several days, reaching maximal mechanical allodynia at about seven days post-surgery (as measured with the von Frey test) [22,29]. Typically, allodynia is detected in the ipsilateral operated paw without affecting, or with minimum impact, on the response of the contralateral paw. Later on, the animals slowly recover their pre-surgery responses. By contrast, the control sham-operated mice only show mild changes in their response to nociceptive stimuli. Notably, disruption of the *HINT1* gene enhances the pain syndrome relative to the 129 WT mice and most significantly, the contralateral paw also sensitizes, as witnessed by the pain-associated responses in the von Frey test. Conversely, disruption of the *σ1R* gene in CD1 mice averts mechanical allodynia (Figure 3).

**Figure 4:**
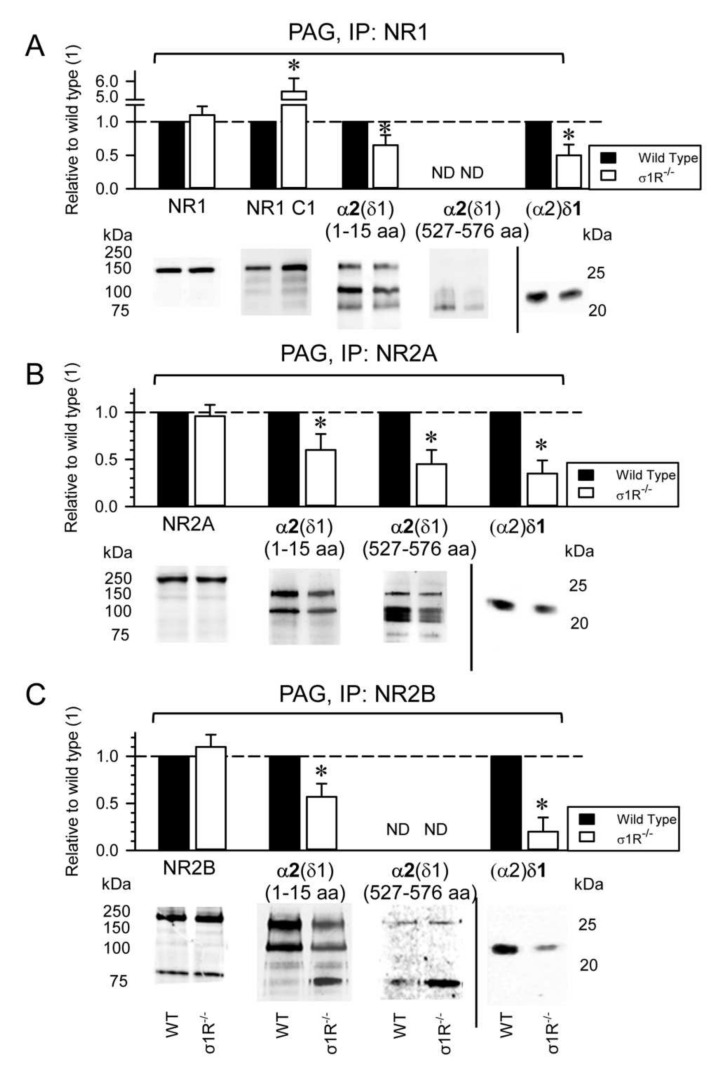
Influence of σ1Rs on the presence of NMDAR-α2δ1-2 complexes. CD1 WT (*n* = 9) and CD1 σ1R^−/−^ (*n* = 9) mice were sacrificed, and PAG synaptosomal fractions were prepared and solubilized. (**A**) NR1, (**B**) NR2A, and (**C**) NR2B subunits were immunoprecipitated (IP) from PAG solubilized proteins. Co-precipitated α2δ1 protein was detached from the NMDAR bait subunits and the presence of α2(δ1) and (α2)δ1 was analyzed in Western blots. The bars represent the mean ± SD from three assays carried on tissue obtained from different groups of three mice. Data from the CD1 σ1R^−/−^ mice are relative to the CD1 WT control, which was assigned an arbitrary value of 1: * indicates significant differences relative to the WT control group, *p* < 0.05 (representative Western blots are shown, see Section 2 for further details). ND: not determined because of a weak signal.

These observations suggest a role for the HINT1 and σ1R proteins in the development of NMDAR-mediated neuropathic pain. The association of the NR1, NR2A, and NR2B NMDAR subunits with α2δ1 proteins was evaluated in CD1 mice lacking σ1R, and in 129 mice devoid of HINT1 proteins. In the absence of CCI, the PAG of WT CD1 and σ1R^−/−^ CD1 mice exhibited comparable levels of NR1, NR2A, and NR2B subunits (Figure 4A–C), although there was a strong increase of the NR1 C1 variant in σ1R^−/−^ mice (Figure 4A) as reported previously [13,25]. Because total NR1 levels were similar in WT and σ1R^−/−^ mice, NR1 subunits lacking the C1 cytosolic region (i.e.,: NR1 C0-C2(2′)) were expected to diminish in σ1R^−/−^ mice. While the levels of NR2A and NR2B subunits were comparable between PAG of WT CD1 and σ1R^−/−^ CD1 mice, the association of α2δ1 proteins with the NR1 and NR2A/B subunits diminished in σ1R^−/−^ mice (Figure 4).

The antibody directed against the N terminal sequence of the α2(δ1) protein detected its association with the NR1, NR2A, and NR2B subunits, whereas the antibody directed against the internal α2(δ1) sequence (aa 527–576) mainly detected an association of α2(δ1) with NR2A (Figure 4B), but not with the NR1 or NR2B subunits (Figure 4A,C). Both antibodies, against the N terminal and internal sequence, labelled bands of about 75 kDa, which may be due to degradation of the α2(δ1) proteins (Figure 4). Presumably, glycosylation of the α2(δ1) 527–576 internal sequence differs between the α2(δ1) proteins that interact with the NR2A subunits and NR1/2B subunits, with the sugars of those α2(δ1) proteins bound to NR1/2B blocking the access of the antibody to the target epitope (Figure 4A,C, Supplementary Material Figures S4 and S5).

**Figure 5:**
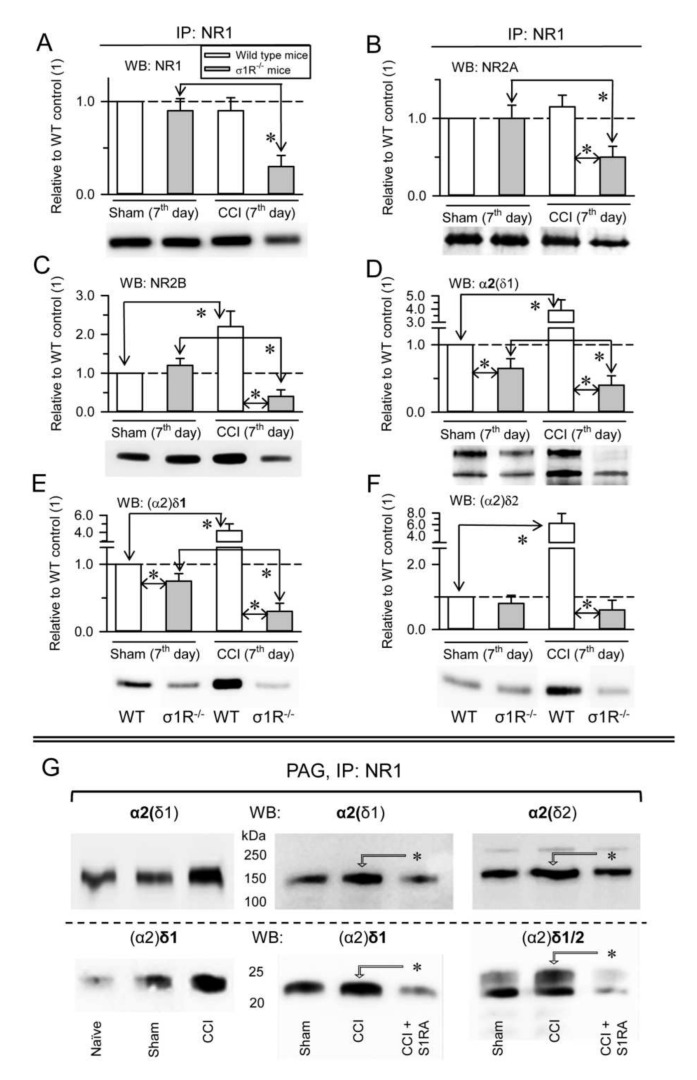
CCI promotes σ1R-mediated associations of α2δ1-2 proteins with NR1 subunits. (**A**) CD1 WT sham-operated (*n* = 4), CD1 WT CCI (*n* = 4), CD1 σ1R^−/−^ sham (*n* = 4) and CD1 σ1R^−/−^ CCI (*n* = 4) were sacrificed 7 days after surgery and PAG synaptosomal fractions were prepared. NR1 subunits were immunoprecipitated (IP) from the solubilized membrane preparations, and the presence of co-precipitated (**A**) NR1, (**B**) NR2A, (**C**) NR2B, (**D**) α2(δ1), (**E**) (α2)δ1, and (**F**) (α2)δ2 proteins was assessed in Western blots (WB). The bars represent the mean ± SD of three measurements. Data are computed relative to the WT mice and in the absence of CCI (assigned the arbitrary value of 1). The arrows refer to the comparison and * indicates significant difference of the CCI group relative to the CD1 WT or CD1 σ1R^−/−^ group: *p* < 0.05. Details as in Figure 4. (**G**) CD1 WT mice: effect of S1RA on the association of α2δ1-2 proteins with NR1 subunits promoted by CCI (*n* = 6). S1RA (3 nM) was injected icv 7 days after CCI surgery in three CD1 WT mice. The animals were sacrificed 30 min later to obtain the synaptosomal fraction from the PAG. The NR1 subunits were immunoprecipitated from the solubilized membrane preparations, and the co-precipitated α2(δ1), α2(δ2), (α2)δ1, and (α2)δ2 were analyzed in Western blots: * indicating significant difference relative to the CCI group, *p* < 0.05. Sham operated CD1 WT mice (*n* = 3) served as control to the effect of CCI (for further details see Section 2).

In control WT CD1 mice, CCI did not significantly alter the expression of NR1 subunits (Figure 5A) or NR1-associated NR2A subunits (Figure 5B), but it caused an increase in NR1-associated NR2B subunits (Figure 5C), as well as the association of α2δ1 proteins (Figure 5D,E) and of (α2)δ2 peptides with NR1 subunits (Figure 5F). However, in σ1R^−/−^ CD1 mice, this intervention provoked a reduction in the NR1, NR2A, and NR2B subunits (Figure 5A–C), and the presence of α2δ1-NR1 complexes diminished accordingly (Figure 5D,E, Supplementary Material Figure S6). The σ1Rs are involved in central neuropathic pain-related behaviors after mild SC injury in mice [19], and S1RA, a selective antagonist of σ1Rs, prevents and even alleviates the pain caused by nerve injury [16]. Our data indicated that CCI greatly enhanced the association of the α2δ1 and α2δ2 proteins with NMDARs. In these circumstances, the administration of S1RA diminished the association of α2δ1 and α2δ2 proteins with NMDARs to the levels observed in naïve CD1 mice (Figure 5G).

**Figure 6:**
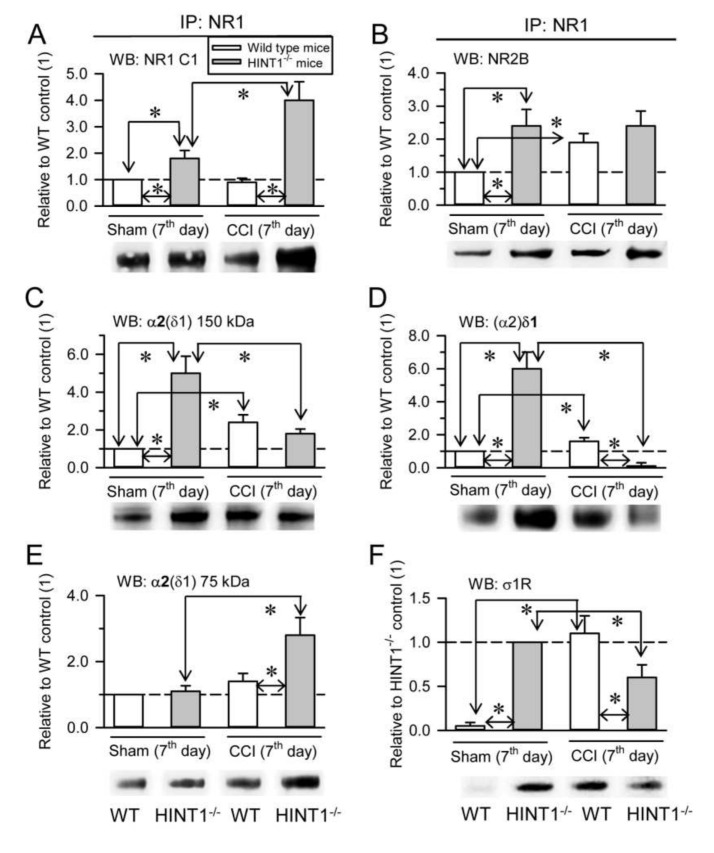
In the absence of HINT1 proteins, CCI promotes the destruction of α2δ1-2 proteins when associated to NMDARs. (**A**) 129 WT sham-operated (*n* = 7), 129 WT CCI (*n* = 7), 129 HINT1^−/−^ sham (*n* = 7), and 129 HINT1^−/−^ CCI mice (*n* = 7) were sacrificed 7 days after surgery to obtain PAG synaptosomal fractions. NR1 subunits were immunoprecipitated (IP) from the solubilized membrane preparations and the presence of co-precipitated (**A**) NR1 subunits carrying cytosolic C1 segment, (**B**) NR2B subunits, (**C**) α2(δ1) proteins of 150 kDa, (**D**) (α2)δ1 peptides, (**E**) α2(δ1) proteins of 75 kDa, and (**F**) σ1Rs were assessed in Western blot (WB). The bars represent the mean ± SD of three measurements and the data are shown relative to the 129 WT sham mice (assigned the arbitrary value of 1). Determination of σ1R: due to the weak signal observed in 129 WT sham mice, the data were assessed relative to the 129 HINT1^−/−^ sham mice (arbitrary value of 1). The arrows refer to the comparison and * indicates significant difference of the CCI group relative to the 129 WT or 129 HINT1^−/−^ control group: *p* < 0.05.

The HINT1 protein also influences the formation of α2δ-NMDAR complexes in PAG synaptosomal membranes as a consequence of nerve injury. We previously reported similar levels of NR1 and NR2A subunits in HINT1^−/−^ 129 mice and their littermate 129 controls, although the NR1 C1 variant and NR2B subunits increased 2-fold in HINT1^−/−^ mice [21]. Our current data confirmed the increase in the NR1 C1 and NR2B subunits, while NR2A subunits in the PAG of HINT1^−/−^ 129 mice did not augment (Figure 6A, Supplementary Material Figure S7A–C). In HINT1^−/−^ 129 mice, NR1 also co-precipitated more NR2B subunits (Figure 6B), and there was an enhanced association of the 150 kDa glycosylated α2(δ1) band and of the (α2)δ1 peptide with NR1 subunits (Figure 6C,D). However, the amount of the α2(δ1) 75 kDa protein remained similar to that in WT 129 mice (Figure 6E). While, σ1R binding to NR1 subunits was readily detected in HINT1^−/−^ 129 mice, this was barely evident in WT 129 mice (Figure 6F). Thus, in the absence of HINT1, the levels of the NR1 C1 variant, the NR2B subunit and the NR1-NR2B association increased, and the σ1R probably augments the association of α2δ1 proteins with NR2B-containing NMDARs. In HINT1^−/−^ 129 mice, NR2A subunits diminished mildly, yet their association with α2δ1 proteins and (α2)δ2 peptides decreased notably (Supplementary Material Figure S7B). By contrast, the levels of NR2B increased, as did their association with the α2δ1 and (α2)δ2 proteins (Supplementary Material Figure S7C).

At seven days after CCI surgery, there was a strong increase of NR1 C1 subunits in HINT1^−/−^ 129 mice, but not in their control littermates (Figure 6A), while there was no change in the association of NR1 with NR2B subunits (Figure 6B). In control 129 mice, CCI increased the association of α2δ1 proteins and σ1Rs with NR1 subunits, yet the strong mechanical allodynia exhibited by HINT1^−/−^ 129 mice was correlated with an important decrease in the association of the α2(δ1) (Figure 6C) and (α2)δ1 (Figure 6D) proteins, and of σ1Rs (Figure 6F), with NR1 subunits, and increases in the α2(δ1) metabolism-related 75 kDa band (Figure 6E). In 129 control mice, CCI surgery increased the total levels of NR2B subunits, but it barely affected those of the NR2A type, and as in CD1 control mice there was an increase in the association of the (α2)δ1 proteins mostly with NR2B subunits (Supplementary Material Figure S7B,C). By contrast, CCI provoked a two-fold increase in the NR2A and NR2B subunits in HINT1^−/−^ 129 mice, although there was a strong reduction in the association of α2δ1 proteins and (α2)δ2 peptides with the NR2B subunits Supplementary Material Figure S7C).

**Figure 7:**
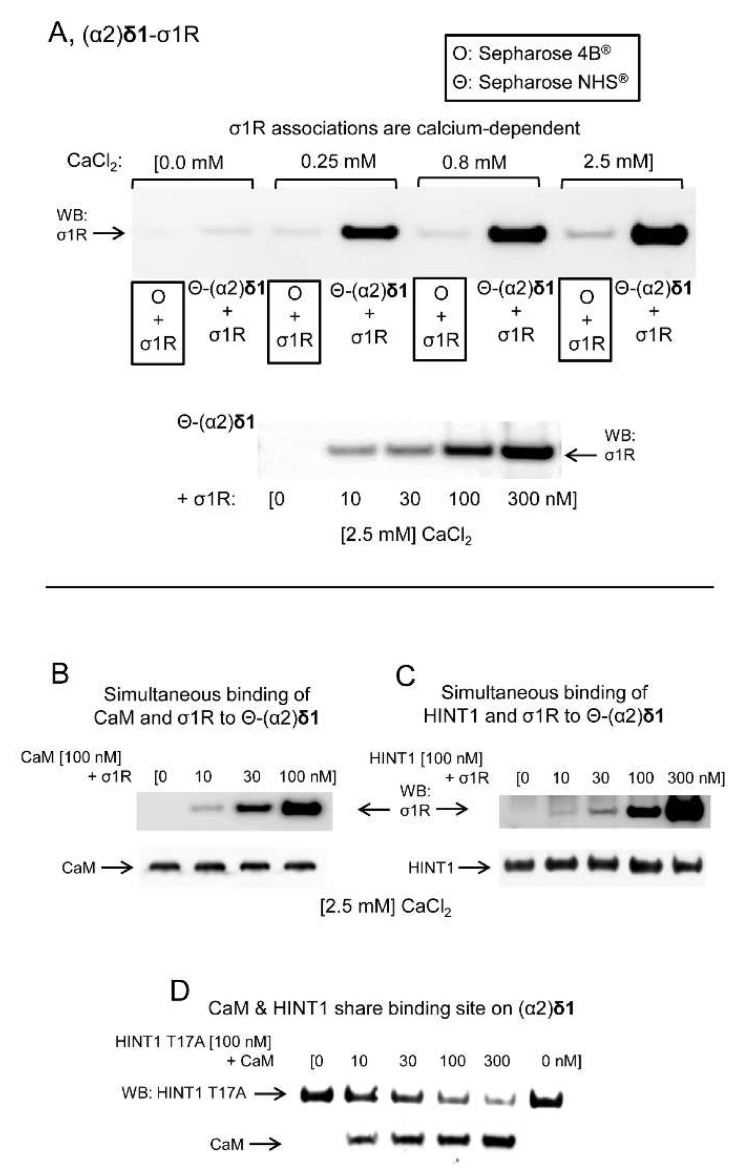
Physical interactions of the (α2)δ1 peptide with σ1Rs, HINT1 proteins, and CaM. (**A**) Calcium-dependent binding of σ1Rs to (α2)δ1 peptides. Recombinant (α2)δ1 peptides covalently attached to NHS-activated Sepharose^®^ were incubated with σ1Rs (100 nM) in the presence of increasing amounts of CaCl_2_. The pellets obtained were washed, solubilized in 2× Laemmli buffer containing β-mercaptoethanol, and resolved by SDS-PAGE. The presence of σ1R was analyzed in Western blots (WBs). The prey protein did not bind to NHS-Sepharose (O + σ1R, negative control). In another set of assays, (α2)δ1 peptides were incubated with increasing concentrations of σ1Rs in the presence of CaCl_2_ (2.5 mM). (**B**–**D**) Competition assays between σ1R, HINT1, and CaM for their binding to (α2)δ1 peptides. CaM (100 nM) was incubated with agarose-(α2)δ1 for 30 min at RT in 300 μL of 50 mM Tris-HCl, [pH 7.5], 0.2% CHAPS, CaCl_2_ (2.5 mM). After removal of the unbound CaM, increasing concentrations of σ1Rs were added. The (α2)δ1-bound proteins were detached, resolved by SDS-PAGE chromatography, and analyzed in Western blots (see Section 2). The assays were repeated at least twice, producing comparable results. This protocol was also used to assess competition between HINT1/σ1R and CaM/HINT1 in their binding to (α2)δ1 peptides. O and Θ represents plain agarose and NHS-Sepharose^®^, respectively.

**Figure 8:**
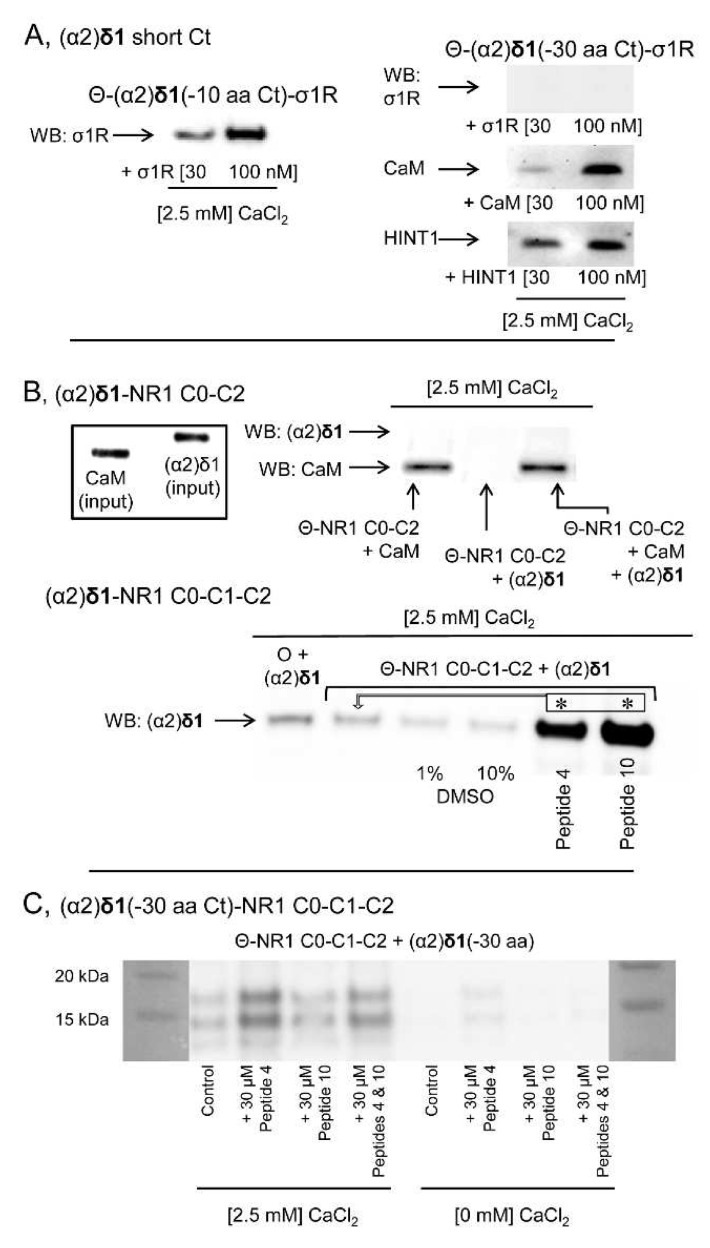
The C terminal sequence of the (α2)δ1 peptide binds to σ1Rs and NMDAR NR1 C1 subunits. (**A**) Immobilized (α2)δ1 C terminal truncated sequences (–10 or –30 aa) were incubated with σ1Rs, CaM or HINT1 proteins (100 nM) in the presence of CaCl_2_ (2.5 mM). The proteins bound to agarose-(α2)δ1 were separated from the unbound fraction by several cycles of washing-resuspension, the bound proteins were detached in 2× Laemmli buffer containing β-mercaptoethanol, resolved by SDS-PAGE and analyzed in Western Blots (WBs). (**B**) The (α2)δ1 peptide binds to the NR1 variant, which contains the cytosolic C1 segment. The cytosolic sequences of the NR1 C0-C2 and NR1 C0-C1-C2 variants (100 nM) were incubated with (α2)δ1 peptides in the presence of CaCl_2_ (2.5 mM). The agarose-bound proteins were detached, resolved by SDS-PAGE and analyzed in WBs. To facilitate the access of (α2)δ1 peptides to the NR1 C1, interactions were performed in the presence of 1 or 10% DMSO, with a peptide (30 μM) mapping to the C0 region (peptide 4, 849–858: QLAFAAVNVW) or the C1 region of the NR1 subunit (peptide 10, 879–888: TFRAITSTLA). The arrows refer to the comparison and * indicates significant difference relative to the control group: *p* < 0.05. (**C**) The peptides mapping to the C0 or C1 cytosolic region of the NR1 subunit did not promote binding of the truncated (–30 aa) (α2)δ1 peptides to the NR1 C1 subunits, either in the presence or absence of CaCl_2_ (2.5 mM: for further details see the Methods). O and Θ represent plain agarose and NHS-Sepharose^®^, respectively.

The possibility that the (α2)δ1 peptide interacts physically with proteins implicated in the regulation of NMDARs was addressed in a series of in vitro assays. The (α2)δ1 peptide bound to σ1Rs in a calcium-dependent fashion (Figure 7A). Calcium-activated calmodulin (Ca^2+^-CaM) [34] inhibits NMDAR activity and like the HINT1 protein it also bound to the (α2)δ1 peptide (Figure 7B,C). The binding of CaM or HINT1 to (α2)δ1 peptides was compatible with that of σ1Rs, although CaM and HINT1 shared a binding site on the (α2)δ1 peptide. This assay was performed with the HINT1 T17A mutant, which fails to bind to CaM [35] (Figure 7D). The removal of the last 30 residues from the (α2)δ1 C terminus abolished its interaction with σ1R. Nevertheless, the cropped (α2)δ1 peptide still bound to CaM or the HINT1 protein (Figure 8A). Ca^2+^-CaM binds to the NR1 variant containing the cytosolic C0-C2(2′) region [34,36], yet to access the NR1 C0-C1-C2(2′) variant, the internal interaction between the C0 hydrophobic region 1 (HR1: 849-858) and the C1 HR2 (879–888) must be annulled. This was achieved experimentally by introducing a peptide complementary to HR1 or HR2 into the incubation medium [24]. While, (α2)δ1 did not bind to NR1 C0-C2, it bound strongly to the NR1 C1 variant in the presence of either of the disrupting peptides (Figure 8B). However, this binding disappeared when the C terminal cropped (−30 aa) (α2)δ1 peptide was used (Figure 8C).

**Figure 9:**
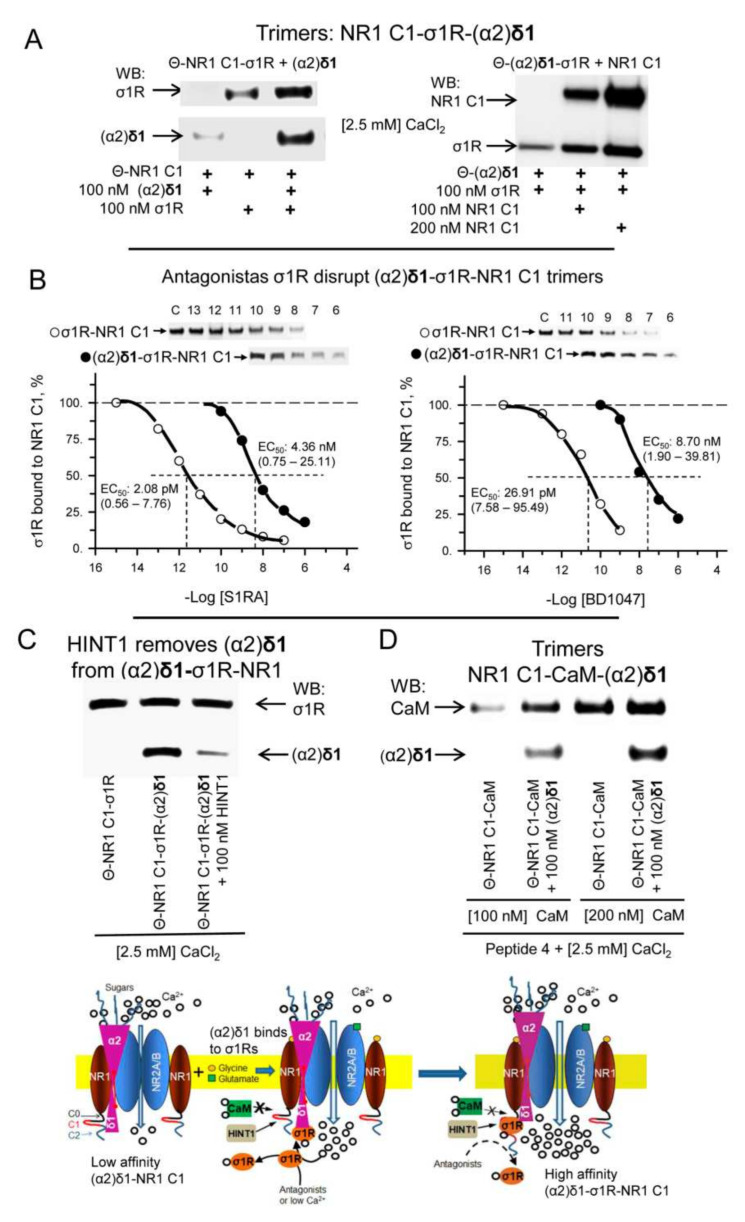
Trimeric associations of (α2)δ1 peptides with σ1R/CaM and NR1 C1 subunits. (**A**) Formation of (α2)δ1-σ1R-NR1 C1 trimers. Agarose(Θ)-attached NR1 C1-σ1R dimers were incubated for 30 min in the presence of (α2)δ1 peptides (100 nM) in 300 μL of 50 mM Tris-HCl, [pH 7.4], 0.2% CHAPS, and CaCl_2_ (2.5 mM). In a set of assays, the NR1 C0-C1-C2 cytosolic region (100 nM) was added to preformed agarose(Θ)-(α2)δ1-σ1R dimers and they were incubated for 30 min at RT. Agarose(Θ)-bound proteins were obtained by centrifugation, washed three times, solubilized in 2× Laemmli buffer containing β-mercaptoethanol, resolved by SDS-PAGE, and analyzed in Western blots (WBs). (**B**) Effect of σ1R antagonists S1RA and BD1047 on (Θ)-σ1R-NR1 C1 dimers and (Θ)-(α2)δ1-σ1R-NR1 C1 trimers. The assays were performed in the presence of 50 mM Tris-HCl, [pH 7.5], 0.2% CHAPS, CaCl_2_ (2.5 mM). S1RA and BD1047 reduced formation of dimers and trimers in a concentration-dependent manner. The data were analyzed by nonlinear regression, competition at a single site (Sigmaplot/Sigmastat v14.5; Systat Software, Inc., Berkshire, UK). S1RA and dimer, −log EC_50_ and 95% confidence interval = 11.68 (12.25–11.11) [2.08 (0.56–7.76) pM]; (estimate ± SE): r = 0.979 ± 0.085, DF total = 7, MS = 0.130, *p* < 0.05, 1 − β = 0.990. S1RA and trimer, −log EC_50_ and 95% confidence interval = 8.36 (9.12–7.60) [4.36 (0.75–25.11) nM]; (estimate ± SE): r = 0.983 ± 0.081, DF total = 5, MS = 0.118, *p* < 0.05, 1 − β = 0.980. BD1047 and dimer, −log EC_50_ and 95% confidence interval = 10.57 (11.12–10.02) [26.91 (7.58–95.49) pM]; (estimate ± SE): r = 0.986 ± 0.077, DF total = 6, MS = 0.153, *p* < 0.05, 1 − β = 0.991. BD1047 and trimer, -log EC_50_ and 95% confidence interval = 8.06 (8.72–7.40) [8.70 (1.90–39.81) nM]; (estimate ± SE): r = 0.986 ± 0.079, DF total = 5, MS = 0.134, *p* < 0.05, 1-β = 0.985. Representative WBs are shown. (**C**) The HINT1 protein removes (α2)δ1 peptides from NR1 C1-σ1R-(α2)δ1 trimers. HINT1 (100 nM) was incubated for 30 min with preformed agarose attached Θ-NR1 C1-σ1R-(α2)δ1 trimers. The agarose pellets were obtained, and the presence of σ1Rs and of (α2)δ1 was determined in WBs. (**D**) CaM forms trimers with NR1 C1 subunits and (α2)δ1 peptides. Since CaM does not bind directly to NR1 C1 subunits, its interaction was facilitated by a peptide mapping to the C0 region (30 μM, peptide 4, 849–858: QLAFAAVNVW). Afterwards, (α2)δ1 peptides were incubated with Θ-NR1 C1-CaM dimers. The presence of CaM and (α2)δ1 in the trimer was subsequently determined in WBs. O and Θ represents plain agarose and NHS-Sepharose^®^, respectively. Inset: Diagram describing the critical role of σ1Rs in the formation of α2δ1-NMDAR complexes. In a resting state, the α2δ1 protein binds to the external surface provided by the interaction between NR1-NR2A/B, with the (α2)δ1 peptide displaying low affinity to the NR1 C1 cytosolic region. The activation of NMDARs by neurotransmitters increases calcium permeation, which recruits σ1Rs and CaM. While σ1Rs gain access to the NR1 C1, CaM does not without the prior binding of certain proteins to this NMDAR region. The formation of the σ1R-NR1 C1 dimer enables (α2)δ1 peptide binding to σ1Rs in the dimer to form the stable (α2)δ1-σ1R-NR1 C1 trimer, which promotes the sustained over activation of NMDARs that leads to neuropathic pain. HINT1 proteins bind to NR1 C1 subunits and diminish the formation of σ1R-NR1 C1 dimers, and by removing (α2)δ1 peptides from the allodynia-related trimer they help control the incidence of this pain syndrome.

As reported above, (α2)δ1 barely binds to NR1 C1 subunits in the absence of disrupting HR1-HR2 peptides, whereas σ1Rs do bind to the NR1 C1 subunits [24]. Importantly, the co-precipitation of (α2)δ1 and σ1Rs with the NR1 C1 subunits increased when they were incubated together. These trimers were formed by incubating NR1 C1 with σ1Rs and after removing the free receptors, NR1 C1-σ1R dimers were exposed to (α2)δ1 peptides. The incubation of σ1Rs with (α2)δ1 peptides produced σ1R-(α2)δ1 dimers that also formed (α2)δ1-σ1R-NR1 C1 trimers in the presence of the NR1 C1 subunits (Figure 9A). The formation of (α2)δ1-σ1R dimers and of (α2)δ1-σ1R-NR1 C1 trimers was highly dependent on calcium and notably, dimer formation diminished greatly after removing calcium from the medium, although the trimer mainly persisted (Supplementary Material Figure S8A). Antagonists of σ1Rs disrupt the in vitro interaction of this receptor with NR1 C1 subunits [24,37] and thus, we evaluated the activity of these drugs to disrupt the existing (α2)δ1-σ1R-NR1 C1 trimers. The EC_50_ values for σ1R antagonists like S1RA and BD1047 to reduce σ1R-NR1 C1 dimers by half was about 2 and 27 pM, respectively, whereas 4 and 8 nM of these drugs was necessary to reduce the presence of (α2)δ1-σ1R-NR1 C1 trimers by 50% (Figure 9B). Agonists of σ1Rs like PRE084 and Pregnenolone sulfate did not affect the formation of σ1R-mediated trimers (Supplementary Material Figure S8B), and agonists and antagonists of σ1Rs failed to disrupt the (α2)δ1-σ1R complexes (Supplementary Material Figure S8C).

There is some interplay between HINT1 proteins and (α2)δ1 peptides. The HINT1 protein gains access to NR1 C1 subunits with little dependence on calcium. However, in the absence of calcium, (α2)δ1 disrupted HINT1-NR1 C1 binding, and while (α2)δ1 still dissociated the HINT1-NR1 C1 complex in the presence of calcium, the peptide now remained bound to the NR1 C1 subunits. This observation indicated that HINT1 facilitates access of the (α2)δ1 peptide to NR1 C1, and that HINT1 and this peptide share a binding site in the C0-C1-C2 cytosolic tail (Supplementary Material Figure S8D). Although, σ1R ligands do not disrupt (α2)δ1-σ1R complexes, HINT1 proteins removed (α2)δ1 peptides from the σ1R-NR1 C1 dimers. Thus, HINT1 increases the efficacy of σ1R antagonists and of low calcium levels to remove σ1Rs from NMDAR NR1 C1 subunits (Figure 9C). In assays in which the NR1 HR1-HR2 interaction was disrupted, Ca^2+^-CaM binding to NR1 C1 subunits enhanced that of (α2)δ1 peptides (Figure 9D). Because the (α2)δ1 peptide did not bind to Ca^2+^-CaM-NR1 C0-C2, the possibility exists that rather than Ca^2+^-CaM binding to the (α2)δ1-NR1 complex both proteins bind simultaneously to different regions of the NR1 C0-C1-C2 cytosolic tail. Analysis of the (α2)δ2 peptide revealed interactions with σ1Rs and CaM (Supplementary Material Figure S9A,B) but not with HINT1 proteins (Supplementary Material Figure S9C). Moreover, when the NR1 C1 HR1-HR2 interaction was disrupted, the (α2)δ2 peptide bound to this NMDAR subunit (Supplementary Material Figure S9D).

**Figure 10:**
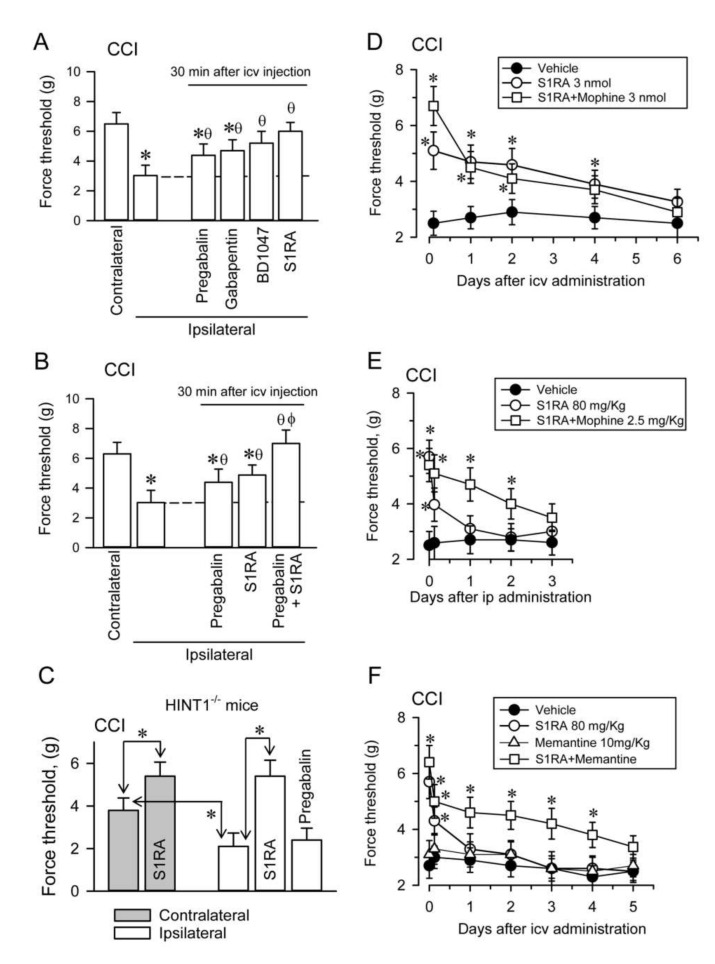
Effect of σ1R ligands and gabapentinoids on the mechanical allodynia displayed by mice. Paw withdrawal thresholds were measured 7 days after CCI surgery and the force (in grams) at which the mice withdrew their paws in response to von Frey hair stimulation was determined as an index of mechanical allodynia. Pharmacological interventions were performed 7 days after CCI. (**A**) Anti-allodynia compounds (3 nmol) were injected intracerebroventricularly (icv) into CD1 WT mice and their effect was evaluated 30 min later. The dashed line indicates the allodynic effect on the ipsilateral paw: * significantly different relative to the contralateral paw; θ indicates significantly different relative to the ipsilateral paw treated with vehicle (groups of 8 mice each). (**B**) CD1 WT mice. Synergic effect of pregabalin (2 nmol, icv) and S1RA (1 nmol, icv) on CCI-induced mechanical allodynia. * Significantly different relative to the contralateral paw; θ significantly different relative to the ipsilateral paw treated with vehicle; ɸ significantly different relative to the ipsilateral paw treated with pregabalin or S1RA (groups of 8 mice each). (**C**) S1RA and pregabalin (3 nmol, icv) were administered to 129 HINT1^−/−^ mice and mechanical allodynia was evaluated 30 min post-injection: * indicates significant differences (groups of 8 mice each). (**D**) The σ1R antagonist S1RA was administered icv to CD1 WT mice alone or 30 min before morphine icv, and the nociceptive threshold was evaluated at the post-injection intervals indicated. At each time interval,* indicates significantly different relative to the vehicle (groups of 6 mice each). (**E**) S1RA was administered by the intraperitoneal (ip) route to CD1 WT mice, alone or 30 min before morphine ip, and the nociceptive threshold was evaluated at the post-injection intervals indicated. * Significant differences relative to the vehicle control group at each time interval (groups of 7 mice each). (**F**) Memantine, a non-competitive NMDA antagonist, was administered to CD1 WT mice ip, alone or together with S1RA, and the nociceptive threshold was evaluated at the post-injection intervals indicated.* Significant differences relative to the nociceptive threshold of the control group at each time point (groups of 6 mice each). All data are presented as the mean ± SD and all the data were analyzed by pairwise Holm-Sidak multiple comparison tests following ANOVA: *p*  <  0.05, 1 − β > 0.80.

The administration of gabapentinoids like pregabalin and gabapentin by icv route diminished the mechanical allodynia in CCI CD1 mice, although σ1R antagonists BD1047 and S1RA had a more potent effect in this sense (Figure 10A). In CCI WT CD1 mice, doses of pregabalin and S1RA that produced moderate effects abolished allodynia when combined (Figure 10B). These effects were correlated with a reduced association of α2δ1 proteins with NR1 subunits in PAG and SC synaptosomes (Supplementary Material Figures S10 and S11). In CCI HINT1^−/−^ 129 mice, S1RA icv diminished the incidence of allodynia but pregabalin promoted no such positive effect (Figure 10C).

Administration of S1RA to CCI WT CD1 mice by the icv route diminished the mechanical allodynia observed in the ipsilateral paw for at least four days, suggesting the relevance of supraspinal regulation in the control of this peripheral pain syndrome. In this scenario, the MOR agonist morphine (3 nmols), barely alleviated neuropathy. The icv administration of S1RA 30 min before that of icv morphine increased the anti-allodynia effect of S1RA during the first hour; afterwards, the σ1R antagonists displayed its own effect (Figure 10D). In CCI WT CD1 mice, the effect of administering S1RA by the intraperitoneal (ip) route was much weaker than when it was administered icv, and its positive effect on allodynia disappeared after a few hours. Morphine (2.5 mg/Kg), only alleviated neuropathy for a couple of hours. However, the systemic administration of S1RA 30 min before that of morphine strongly diminished neuropathic pain for at least two days (Figure 10E). Importantly, there was some synergy when S1RA and the weak NMDAR antagonist memantine were co-administered systemically to CCI WT CD1 mice, and while S1RA alone alleviated pain for a few hours, memantine did not significantly alter allodynia. Notwithstanding, their combination strongly reduced neuropathic pain for several days, approaching the efficacy of S1RA when administered alone by the icv route (Figure 10F).

## 4. Discussion

In response to nerve injury, the association of α2δ1 proteins with glutamate NMDARs increases, bringing about the onset and maintenance of neuropathic pain. These associations were previously evident at the spinal and DRG level [8]; however, pharmacological interventions at the supraspinal level efficaciously alleviated CCI neuropathy of spinal origin. Thus, our study reports the presence of such neuropathy-related α2δ1-NMDAR associations at the supraspinal PAG level that depend on the interplay between σ1Rs and HINT1 proteins.

In addition to the α2δ1 protein, the α2δ2 variant also binds to NMDARs; however, nerve damage barely induces the appearance of α2δ2-NMDAR complexes at a spinal level, and thus, they may have limited relevance in neuropathy [8,38]. Nevertheless, following spinal CCI there was an increase in α2δ1-NMDAR and α2δ2-NMDAR complexes in the PAG, although the highly selective σ1R antagonist S1RA disrupted both these associations. The α2δ1 and α2δ2 proteins both bind gabapentinoids [12,39], and they form complexes with NR1 C1 subunits. However, while σ1R and CaM bind to the (α2)δ1 peptide in a calcium-dependent fashion, their interaction with the (α2)δ2 peptide is less sensitive to calcium. Moreover, the (α2)δ1 but not the (α2)δ2 peptide binds to the HINT1 protein. It is possible that α2δ2 proteins play a different role in the onset and maintenance of neuropathy, although their regulation in the context of NMDARs may be similar to that of α2δ1 proteins.

The α2δ2 protein is much more strongly expressed in the PAG than in the SC, which might account for the presence of α2δ2-NMDAR complexes in this supraspinal structure. The antibody used in previous studies is directed against the α2(δ2) aa 850–865 sequence, an internal sequence that probably associates with sugars at N864 and that in turn may limit or even abrogate antibody binding. In our study, the polyclonal antiserum directed against a different and longer sequence of α2(δ2) (aa 550–800) revealed this α2δ2-NMDAR interaction. Because α2δ proteins are heavily glycosylated [3,6], the presence of sugars associated with their peptide sequence or even the variable sugar decoration at the same sequence, makes immunodetection complicated. In fact, the antibody directed against the internal sequence aa 527–576 of the α2(δ1) protein only detected the targeted protein when bound to NR2A, but not to NR2B subunits. Thus, the N terminal 1–15 α2(δ1) antibody labelled α2δ1 proteins associated with NR1, NR2A, and NR2B subunits in the absence of nerve injury, whereas the 527–576 α2(δ1) antibody mainly detected the association of α2(δ1) with NR2A but not with the NR1 or NR2B subunits. The CCI procedure enhanced the α2δ1-NMDAR associations, but again, the 527–576 α2(δ1) antibody indicated that the α2δ1 proteins bound to NR1 subunits are essentially contributed by NR2B subunits. This observation is consistent with previous reports suggesting that NMDARs containing NR2B subunits are those involved in neuropathic pain [40,41].

Glycosidase enzymes diminished the apparent size of the α2δ proteins [3,6] and accordingly, we found that PNGase F reduced the size of the NR2A/B-associated α2(δ1) proteins from 150 to 100 kDa. In the ER, glycosylation introduces a signal for protein membrane localization or exocytosis and in the particular case of α2δ, this modification is required for the functional membrane expression of calcium channels. Indeed, deglycosylation and glycosylation site-directed mutagenesis strongly reduces current densities without affecting the kinetic properties of such channels [6,42]. Therefore, the disparate glycosylation of α2δ1 proteins associated with NR2A or NR2B subunits may also modify their influence on NMDAR activity.

In cell expression systems, α2δ1 proteins bind to heterodimers of NR1 with NR2 subunits, but not to NR1, NR2A, or NR2B when expressed alone [8]. Thus, the NR1-NR2 dimer offers a binding surface to the extracellular α2(δ1) protein and to the transmembrane/cytosolic C terminal (α2)δ1 peptide, the latter being critical to stabilize the interaction. Remarkably, (α2)δ1 binds to the NR1 variant, which contains the cytosolic C1 segment intercalated between the C0 and C2(2′) regions, displaying no affinity towards the NR1 C0-C2(2′) variant. This preference is evident with GPCRs, which interact through their cytosolic C-terminus with NMDAR NR1 C1 subunits [25,43,44], and also in the tandem σ1R-HINT1 proteins [24,45], which connect GPCRs like the MOR and CB1R to NMDARs [25]. Thus, an external surface in the NMDAR provided by the NR1 C1 subunit when coupled to NR2A or NR2B subunits physically interacts with α2δ1 proteins. Collectively, these observations suggest an important role for NMDARs that contain NR1 C1 subunits in the impact of signals originated at GPCRs. In fact, NR1 C1 subunits are enhanced in depressive patients and they diminish in those affected by schizophrenia [46], augmenting five-fold in σ1R^−/−^ mice and about two-fold in HINT1^−/−^ mice. These changes do not affect the total NR1 levels, but they are compensated by fluctuations in the content of the C0-C2(2′) variant [21,25].

In the absence of nerve injury, the association of α2δ1 proteins with NMDARs does not promote noticeable neuropathic pain. Thus, HINT1 or (α2)δ1 at NR1 C1 subunits would barely alter the activity of NMDARs when triggered by regulators, such as glutamate, glycine, or D-serine. Enhanced GPCR signaling as a consequence of nerve lesion recruits PLCβ to activate PKCγ. This kinase acts on the NR1 C1 segment [22] to exchange HINT1 binding with that of σ1Rs, which now facilitates (α2)δ1 access to NR1 C1 subunits and stabilizes the α2δ1-σ1R-NMDAR interaction, augmenting calcium permeation [8,24]. HINT1 proteins and σ1Rs compete for binding to NR1 C1 subunits in a calcium-dependent manner. Thus, in the absence of nerve injury, the interplay between HINT1 proteins and σ1Rs determines the extent of NMDAR activation. A single NMDAR contains two NR1 subunits, which may be different variants: C0-C1-C2(2′) or C0-C2(2′). In this case, NMDARs exhibit intermediate deactivation kinetics and pharmacological properties compared to the respective NR1-NR2A/B or NR1 C1-NR2A/B receptors [47]. Thus, activation promoted by σ1Rs at C0-C1-C2 may be counterbalanced by inhibitory Ca^2+^-CaM at C0-C1 [36]. Regulation of NMDAR activity is also achieved by endogenous ligands of the σ1Rs. Agonists promote and antagonists dampen σ1R-NR1 C1 interactions that regulate the access of HINT1 proteins and of Ca^2+^-CaM to NR1 C1 subunits, thereby influencing the open probability of the NMDAR pore [24].

The (α2)δ1 peptide binds to the HINT1 protein, CaM, σ1R, and the NR1 C1 subunit. HINT1 and CaM at least partially, share their binding site on the (α2)δ1 peptide, and the σ1R and NR1 C1 bind to the last 30 aa of the (α2)δ1 C terminus. Thus, the (α2)δ1 peptide may also associate with a number of regulatory proteins so that they are immediately available when needed in the NMDAR compartment. Nerve damage augments the signaling activity of certain GPCRs [48], providing σ1Rs to bind to NR1 C1 subunits [24], sustaining calcium permeation and thus, the calcium available at the cytosolic side of the NMDAR pore. This mechanism promotes two opposing signaling pathways, the increase in Ca^2+^-CaM drives the release of HINT1 proteins from (α2)δ1 peptides to diminish the access of σ1Rs to NR1 C1 subunits and thus, NMDAR activity. However, σ1Rs can also bind to the (α2)δ1 C terminal region forming (α2)δ1-σ1R-NR1 C1 trimeric complexes that protect NMDAR activity. The number of these trimers would increase as the activity of PKCγ releases more of the HINT1 bound to NR1 C1 subunits [22,24], making these NMDAR subunits available to interact with (α2)δ1-σ1R.

In vitro, the trimer is more stable than the dimer when calcium levels decrease or when compared to the dissociative effect of σ1R antagonists. In this scenario, the trimer promotes NMDAR over activation and provokes the ensuing mechanical allodynia. This pro-nociceptive situation can be alleviated by HINT1 proteins removing (α2)δ1 peptides from the σ1R-NR1 C1 dimer, thereby increasing the dissociation of σ1R-NR1 C1 complexes as calcium diminishes or in the presence of σ1R antagonists. The (α2)δ1-NR1 C1 association observed in the absence of nerve damage/neuropathy may be mediated by CaM maintaining the NMDAR inhibitory (α2)δ1-CaM-NR1 C1 trimer even at low calcium levels. In this situation, the mobilization of HINT1 proteins would remove (α2)δ1 peptides following CaM separation from NR1 C1 at low calcium.

While CCI did not alter the NR1 C1 variant content in CD1 WT and 129 WT mice, NR2B subunit expression augmented and thus, there was an increase in the α2δ1 protein binding to NMDARs through NR2B subunits. The expression of total NR1 is similar in CD1 WT and CD1 σ1R^−/−^ mice, but in the latter, the NR1 C1 variant increases about five-fold [25]. Nevertheless, this increase does not facilitate access of α2δ1 proteins to NMDARs and thus, HINT1 binding to NR1 C1 subunits augments [24,25]. By increasing NR1 C1 subunit expression, CD1 σ1R^−/−^ mice may at least partially restore the interaction between GPCRs and NMDARs. These associations are facilitated by σ1Rs and they are further reduced by HINT1 transfer from GPCRs towards NMDARs in CD1 σ1R^−/−^ mice [25], favoring the formation of HINT1-NR1 C1 dimers. As mentioned, nerve injury does not promote α2δ1-NMDAR associations or cause mechanical allodynia in CD1 σ1R^−/−^ mice. In these mutant mice, (α2)δ1 peptides may be switched with HINT1 proteins at NR1 C1 subunits, yet such (α2)δ1-NR1 C1 complexes apparently exert no significant effect on NMDAR activity. Thus, σ1Rs certainly appear to be decisive to form neuropathy-related (α2)δ1-σ1R-NR1 C1 complexes.

HINT1 proteins couple weakly active NMDARs to certain GPCRs, such as MORs. In this context, the function of the GPCR activates the coupled NMDAR, which now separates to negatively regulate the signaling of the GPCR. In 129 mice with a targeted deletion of the *HINT1* gene, GPCRs lack this negative feedback and thus, NR1 C1 levels may increase to restore this function. Thus, NR1 C1 and the neuropathy-related NR2B subunit increase two-fold in 129 HINT1^−/−^ mice, and α2δ1 proteins increase their association with these subunits, which influences NMDAR activity [21]. In this mutant mouse, CCI further increases the availability of the NR1 C1 variant and the formation of (α2)δ1-σ1R complexes, although the severe neuropathic syndrome exhibited by these mice [22] was accompanied by a drastic reduction in α2δ1-NMDAR complexes. Because, smaller fragments of α2(δ1) appeared in the PAG of CCI HINT1^−/−^ mice, proteolytic degradation of α2δ1 proteins may account for this reduction.

The binding of HINT1 to NR1 C1 subunits is not very dependent on calcium and σR1s hardly remove HINT1 proteins from NR1 C1 subunits. As mentioned above, this is facilitated by the PKCγ-mediated phosphorylation of the C1 region of NR1 subunits, which reduces the affinity of HINT1 binding to this cytosolic region and increases that of σ1Rs [24]. HINT1 binds in a zinc-dependent manner to cysteine-rich domains in the regulatory region of PKCγ and prevents its kinase activity [49]. Thus, PKC activity is enhanced in the absence of HINT1 [21], facilitating σ1R binding to NMDARs containing NR1 C1 subunits. This mechanism may account for the enhanced mechanical allodynia observed in HINT1^−/−^ mice after CCI surgery.

We have learned how alterations of proteins such as HINT1, σ1R, and NR1 C1 subunit may affect adaptive responses of NMDARs. Indeed, a series of human HINT1 mutants cause autosomal recessive axonal neuropathy with neuromyotonia (ARAN-NM) [50]. In most HINT1 mutants, interactions with a series of signaling proteins are impaired, NR1 C1 and σ1Rs included [51]. Motor neurons are enriched in σ1Rs [52] and autosomal recessive loss-of-function mutations in σ1Rs are primarily associated with distal hereditary motor neuropathy and amyotrophic lateral sclerosis/frontotemporal dementia [53,54]. Thus, HINT1 mutants may promote α2δ1 and σ1R mediated activation of NMDARs, and accordingly, amyotrophic lateral sclerosis could be treated with drugs reducing NMDAR activity [55]. Similarly, there are fewer NR1 C1 subunits in the prefrontal cortex of schizophrenic patients, while they increase in depressive individuals. These changes may alter the cross-talk between GPCRs and NMDARs, and also the capacity of α2δ1 proteins to activate this glutamate receptor [46]. Our present study reveals that in the PAG of CD1 σ1R^−/−^ mice, CCI recruits HINT1 proteins to reduce NMDAR activity, thereby enhancing descending pain control and abolishing the supraspinal perception of neuropathic pain. Alternatively, molecular and electrophysiological studies indicate that 129 HINT1^−/−^ mice exhibit higher NMDAR/AMPAR and NR2B/NR2A subunit ratios [21], and thus, CCI may promote severe σ1R-mediated hypofunction of PAG glutamate activity, which compromises descending pain control and enhances the supraspinal impact of allodynia [27].

The HINT1 protein reduces the formation of pro-allodynic (α2)δ1-σ1R-NMDAR complexes and thus, neuropathy is enhanced in the absence of HINT1. In this scenario, α2δ1 proteins undergo proteolysis, probably in an attempt to reduce the impact of pain mediated by NMDAR overactivity. Unfortunately, proteolysis of α2δ1 proteins may remove the gabapentinoid binding site from α2(δ1), and in fact gabapentinoids do not alleviate allodynia in 129 HINT1^−/−^ mice. This phenomenon may account for the large number of patients suffering neuropathy who are refractory to the beneficial effects of α2(δ1)-binding gabapentinoids, almost 50% [56]. Thus, selective σ1R antagonists may be the agents of choice to treat gabapentinoid-resistant neuropathy. The efficacy of systemic S1RA increases considerably when combined with morphine; however, this potentiation is not observed when both compounds are administered via the icv route. Hence, spinal MORs would appear to be more relevant than brain MORs in reducing CCI-induced neuropathy. Thus, at the supraspinal level, S1RA may collaborate with activated spinal MORs to alleviate neuropathic pain of spinal origin. Notably, anti-allodynia evoked by systemic administration of the σ1R antagonist S1RA is enhanced and it persists for longer when combined with low doses of memantine, a low affinity antagonist of NMDARs. Because NMDARs containing NR2B subunits are critical to regulate peripheral persistent inflammatory pain [57], NR2B specific antagonists may also alleviate mechanical allodynia.

In summary, our study suggests that the α2δ1-NMDAR association, and hence allodynia, depends on the interplay between σ1Rs and HINT1 proteins. Interestingly, recent reports suggest a potential therapeutic role for exogenous regulators of σ1R and HINT1 in the clinical management of neuropathic pain [17,22]. The possible use of such pharmacological interventions to alleviate the progression of this pain syndrome merits consideration.

## 5. Conclusions

- Nerve damage recruits σ1Rs, which couple α2δ1 proteins to NR1 subunits, enhancing NMDAR activity and neuropathy.
- The formation and stabilization of the σ1R-NR1 dimers depends on calcium, and they can be dissociated by σ1R antagonists. By contrast, neuropathy-related δ1-σ1R-NR1 trimers remain stable even in the presence of low levels of calcium, and they are much less sensitive to the effect of σ1R ligands.
- The binding of HINT1 proteins to NR1 subunits does not require calcium and it limits the access of σ1Rs to NMDARs. In addition, once the δ1-σ1R-NR1 trimer forms, HINT1 removes the δ1 peptide and restores the potential of antagonists to disrupt the σ1R-NR1 interaction.
- Thus, σ1Rs and HINT1 proteins control the access of α2δ1 proteins to NMDARs, their activating capacity, and consequently, the severity of neuropathic pain syndrome.

## Data Availability

No new data were created or analyzed in this study. Data sharing is not applicable to this article.

## Supplementary Materials

The following are available online at https://www.mdpi.com/article/10.3390/biom11111681/s1, Figure S1: Expression of α2(δ1), (α2)δ1, and (α2)δ2 in the cerebral cortex, PAG, pons-medulla, and spinal cord of CD1 wild type and σ1R^−/−^ mice, Figure S2: Expression of α2(δ1), (α2)δ1, and (α2)δ2 in the cerebral cortex, PAG, pons-medulla, and spinal cord of 129 HINT1^+/+^ and HINT1^−/−^ mice, Figure S3: Representative Western blots of the assay presented in the Figure 2, Figure S4: Representative Western blots of the assay presented in the Figure 4, Figure S5: Representative Western blots of the assay presented in the Figure 4 Figure S6: Representative Western blots of the assay presented in the Figure 5, Figure S7: Effect of HINT1 on the molecular associations between the α2δ1-2 and NR1, NR2A or NR2B subunits in the CCI model of neuropathic pain, Figure S8: Effect of increasing concentrations of σ1R agonists, PRE084, and Pregnenolone sulfate, on the NR1 C1-σ1R dimers and NR1 C1-σ1R-(α2)δ1 trimers, Figure S9: The effect of calcium on the physical interactions of (α2)δ2 peptides with σ1Rs, HINT1 proteins, and calmodulin, Figure S10: CCI model of neuropathic pain: the effect of S1RA, BD1047, and gabapentinoids on the molecular associations between the α2δ1 proteins and NR1 C1 subunits in PAG synaptosomes, Figure S11: CCI model of neuropathic pain: the effect of S1RA, BD1047 and gabapentinoids on the α2δ1-NR1 association in spinal cord synaptosomal fraction.
