# Supplementary material for: The σ1 Receptor and the HINT1 Protein Control α2δ1 Binding to Glutamate NMDA Receptors: Implications in Neuropathic Pain
Source: Biomolecules. 2021 Nov 12;11(11):1681. doi: 10.3390/biom11111681 (PMC8615847; doi:10.3390/biom11111681)
Supplement: Supplementary file 1 [file biomolecules-11-01681-s001.zip › biomolecules-1400999-supplementary.pdf]

biomolecules

Supplemental Figures

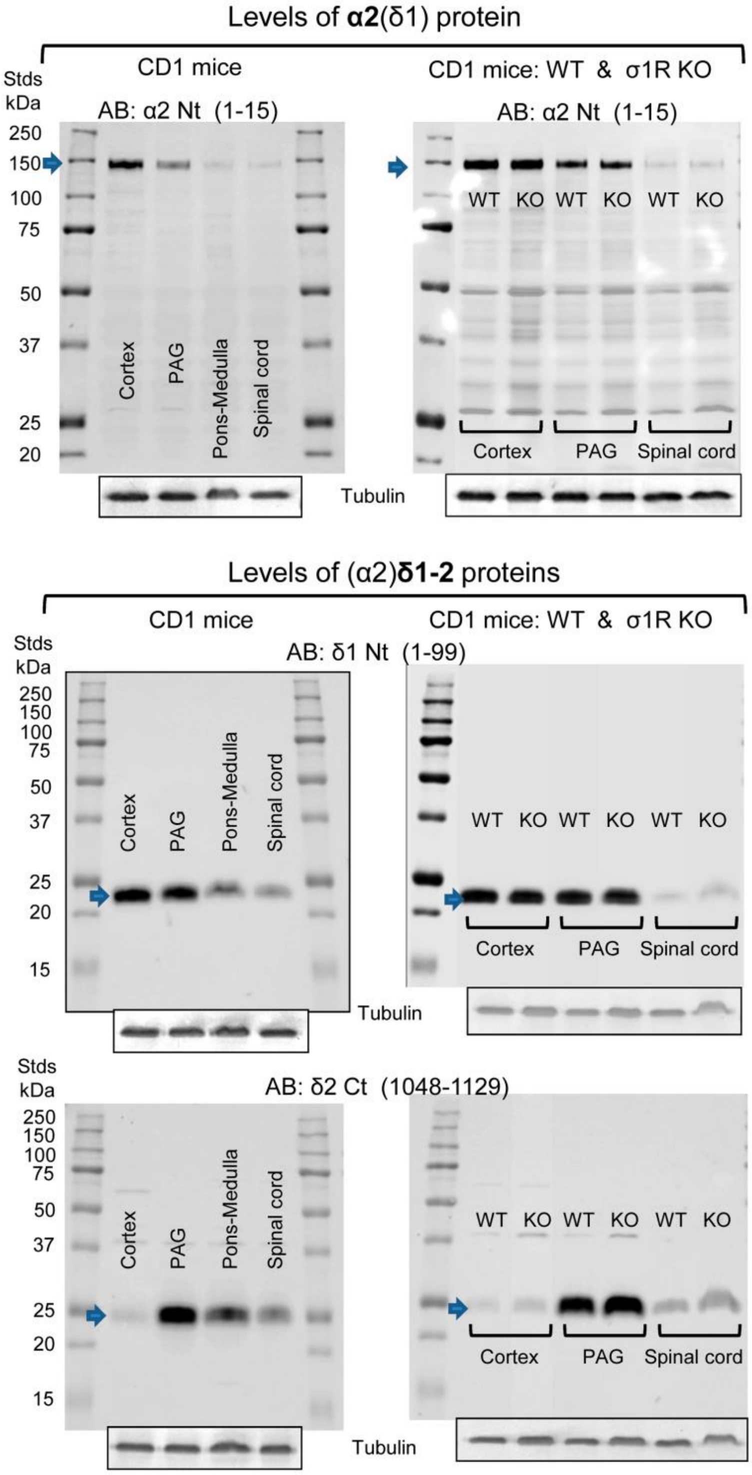

Figure S1. Expression of α2(δ1), (α2)δ1 and (α2)δ2 in the cerebral cortex, PAG, pons-medulla and spinal cord of CD1 wild type and CD1 σ1R$^{-/-}$ **mice.** Mouse neural structures were collected and P2 fractions enriched in synaptosomes were obtained, resolving 60 µg protein/lane by SDS-PAGE and analyzing this extract in Western blots that were probed with antibodies against α2δ1-2 proteins (see Methods, representative Western blots are shown).

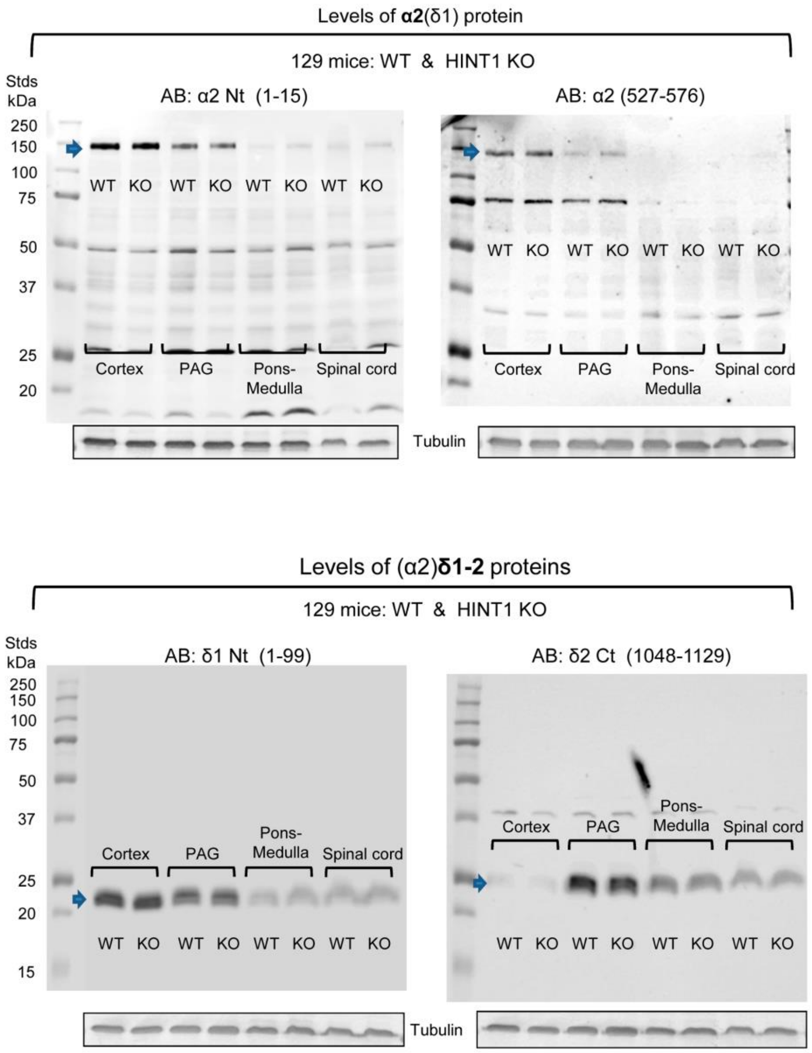

Figure S2. Expression of α2(δ1), (α2)δ1 and (α2)δ2 in the cerebral cortex, PAG, pons-medulla and spinal cord of 129 HINT1+/+ and 129 HINT1-/- mice (for details see Figure S1, representative Western blots are shown).

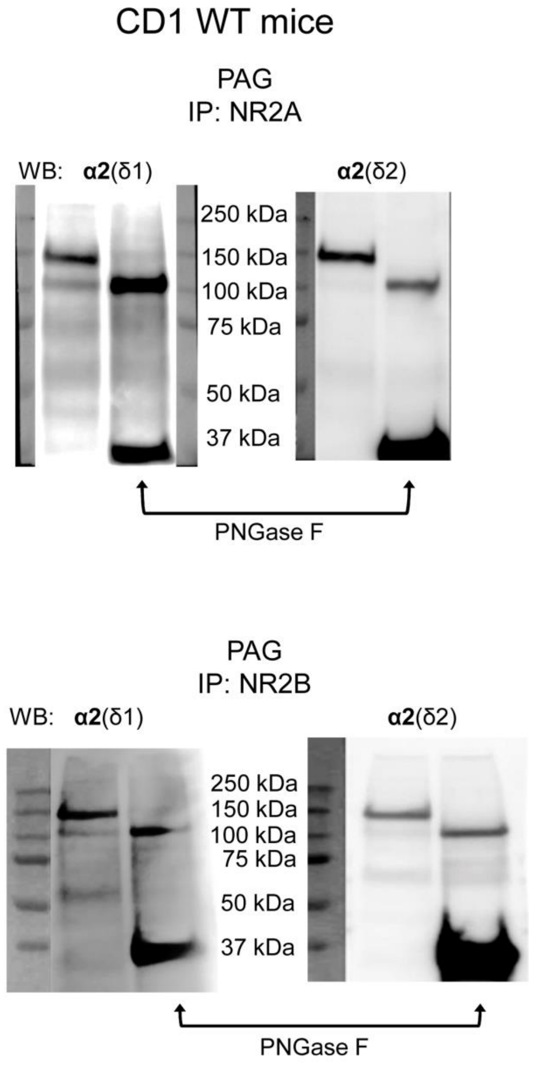

Figure S3. Representative Western blots of the assay presented in the Figure 2 main text.

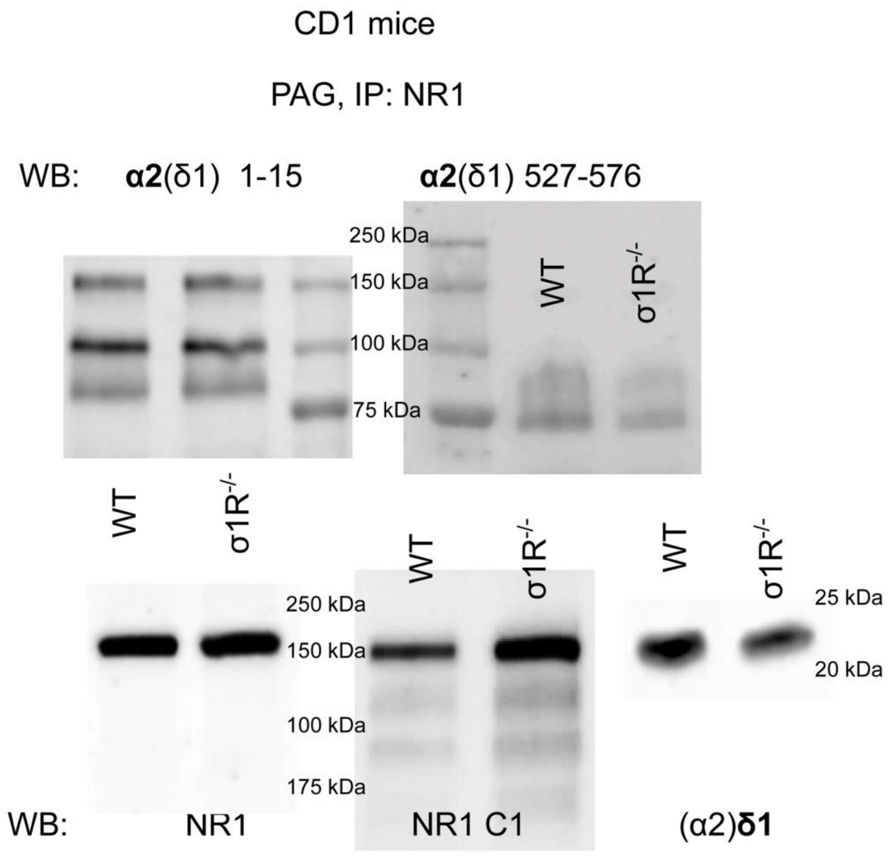

Figure S4. Representative Western blots of the assay presented in the Figure 4 main text.

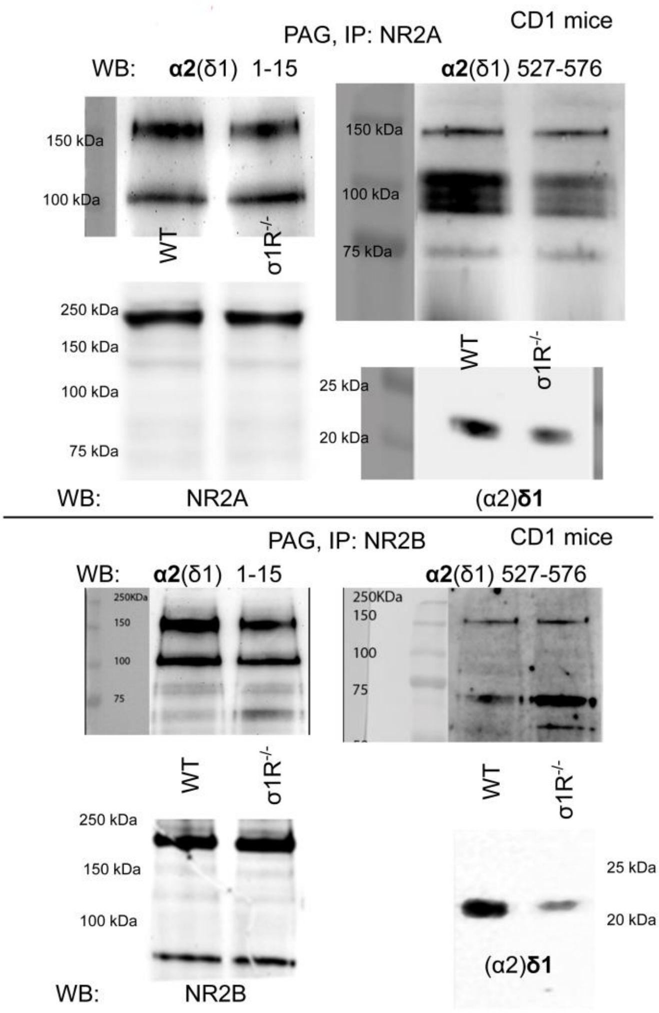

Figure S5. Representative Western blots of the assay presented in the Figure 4 main text.

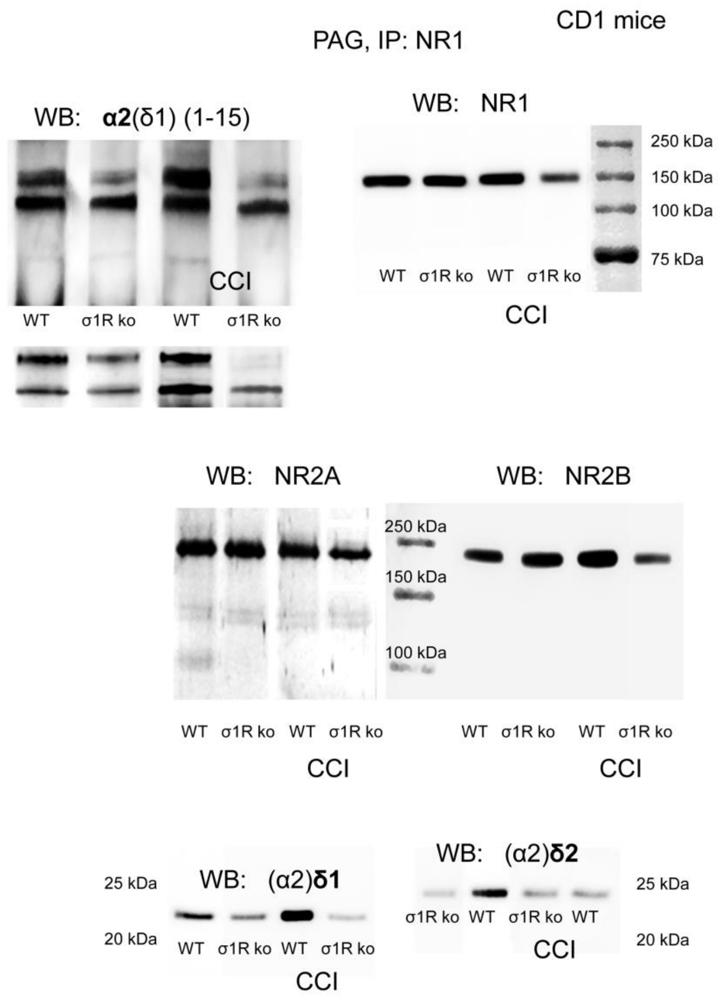

Figure S6. Representative Western blots of the assay presented in the Figure 5 main text.

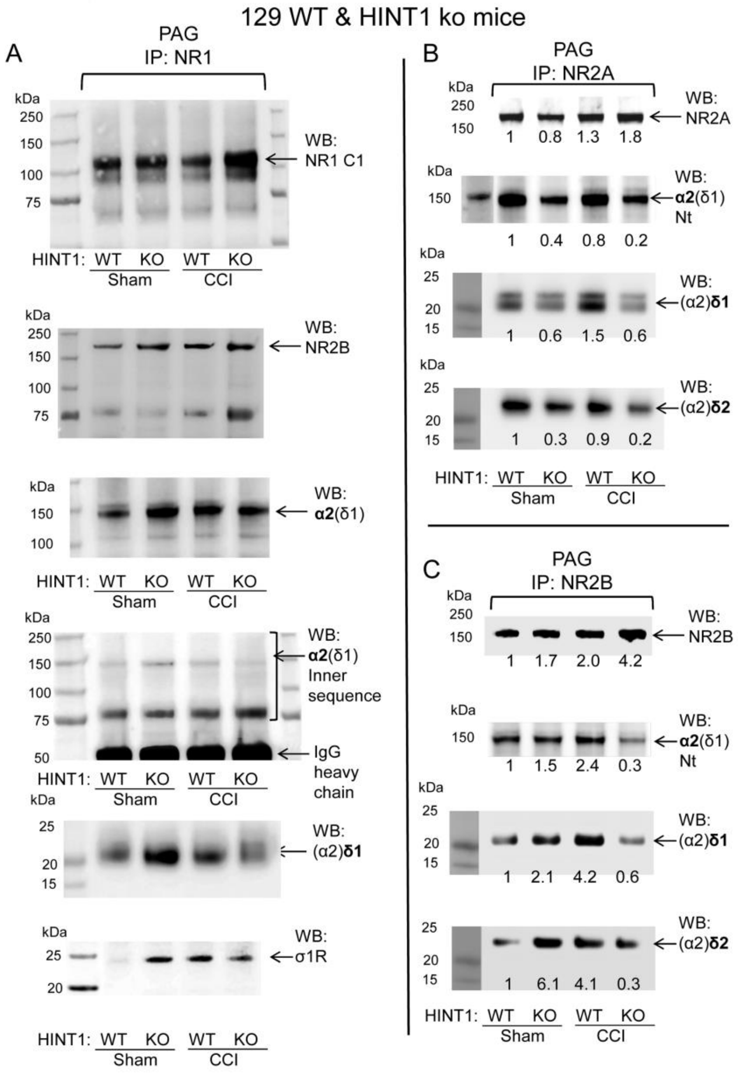

Figure S7. Effect of HINT1 on the molecular associations between the $\alpha 2\delta 1$-2 and NR1, NR2A or NR2B subunits in the CCI model of neuropathic pain. 129 WT and 129 HINT1-/- (KO) mice were sacrificed, and PAG synaptosomal fractions were obtained 7 days after surgery. Following membrane solubilization, (A) NR1, (B) NR2A and (C) NR2B subunits were immunoprecipitated (IP), and the co-precipitated proteins were resolved by SDS-PAGE and then analyzed in Western blots (WB). Representative Western blots are shown (for details see Figure 6).

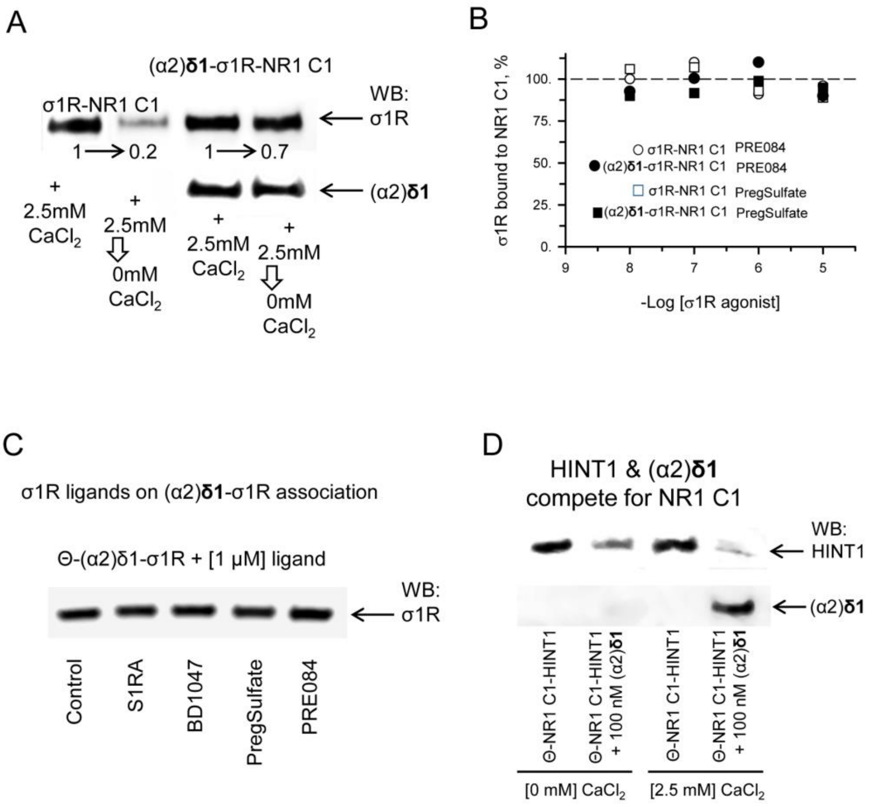

Figure S8. (A) The NR1 C1-σ1R-(α2)δ1 trimer exhibits more stability than the NR1 C1-σ1R dimer at low calcium concentrations. Dimers and trimers were formed in the presence of CaCl2 (2.5 mM) and after removing the calcium by three washes the presence of NR1 C1-bound σ1Rs and (α2)δ1 proteins was determined in Western blots. (B) The influence of increasing concentrations of σ1R agonists, PRE084 and Pregnenolone sulfate, on the NR1 C1-σ1R dimers and NR1 C1-σ1R-(α2)δ1 trimers was assessed. (C) Agonists and antagonists of σ1Rs do not alter the association of σ1Rs with (α2)δ1 peptides. (D) When bound to NR1 C1 subunits, HINT1 proteins can interchange with (α2)δ1 peptides. In the presence or absence of calcium, (α2)δ1 reduces HINT1 binding to NR1 C1 subunits, but in the presence of CaCl2 (2.5 mM), (α2)δ1 remains bound to the NR1 C1 subunit (for details see Figures 7A and 9A).

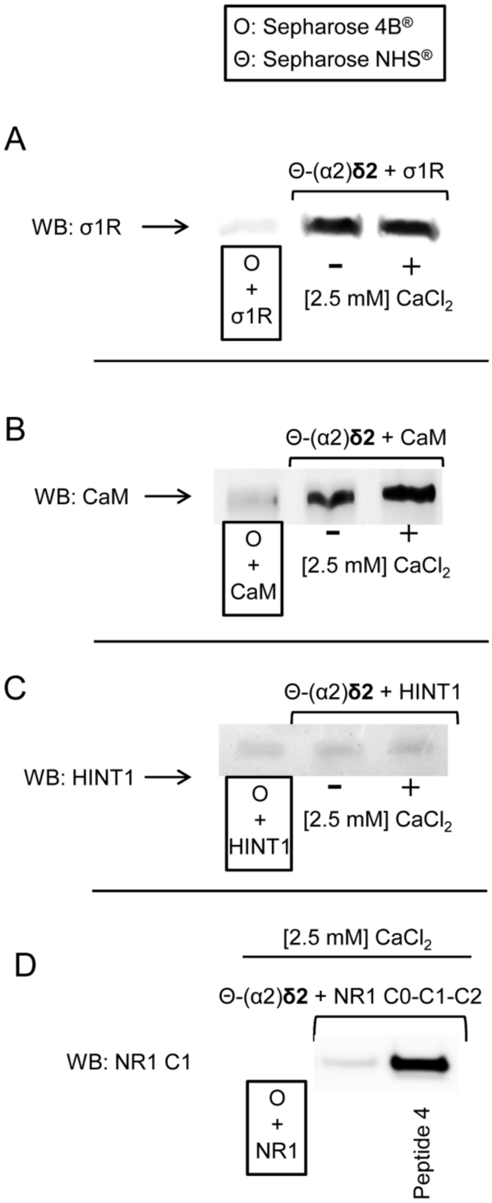

Figure S9. The effect of calcium on the physical interactions of (α2)δ2 peptides with σ1Rs, HINT1 proteins and calmodulin CaM. **A**, The (α2)δ2 peptide binds to σ1Rs in the absence of calcium. **B**, The (α2)δ2 peptide binds to CaM in the absence of calcium. **C**, Calcium does not promote HINT1 protein binding to the (α2)δ2 peptide. **D**, The (α2)δ2 peptide binds to NR1 C1 NMDAR subunits in presence of peptide 4. O and Θ represents plain agarose and NHS-Sepharose®, respectively. For details see Figure 7A.

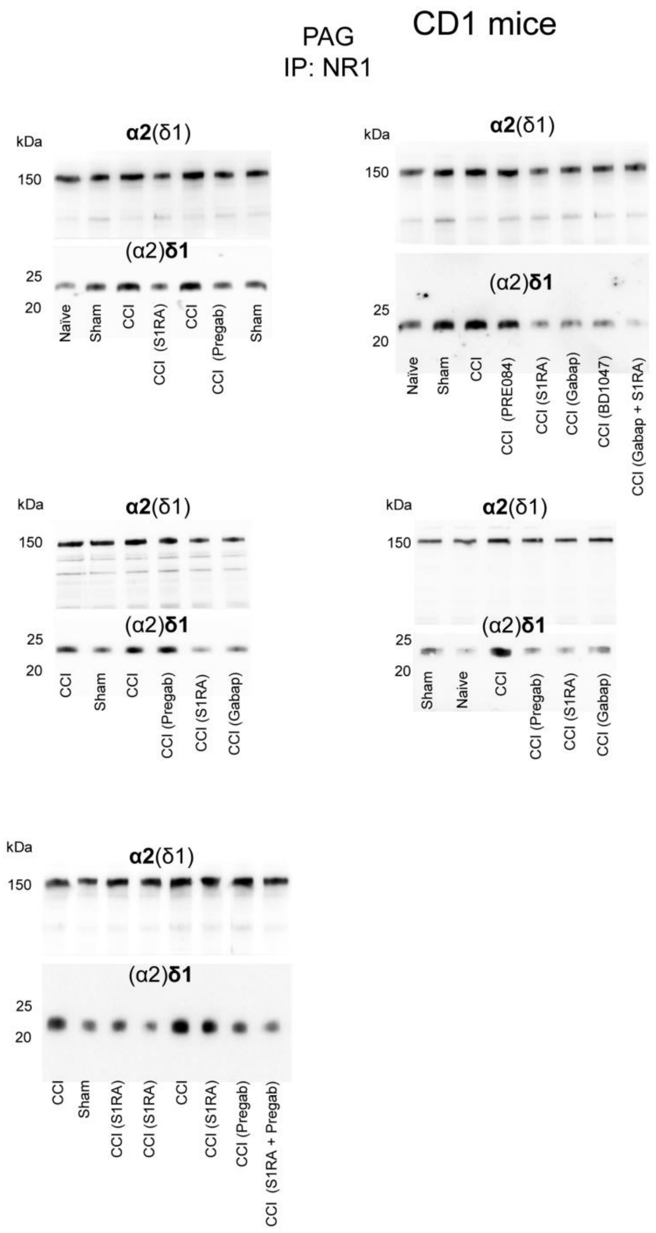

Figure S10. CCI model of neuropathic pain: the effect of S1RA, BD1047 and gabapentinoids on the molecular associations between the $\alpha2\delta1$ proteins and NR1 C1 subunits in PAG synaptosomes. S1RA, Pregabalin, Gabapentin or BD1047 (all 3 nmol) were injected icv into CCI CD1 WT mice 7 days after surgery and after 30 min, the animals were sacrificed and PAG synaptosomal fractions were obtained. The NR1 subunits were immunoprecipitated from the solubilized membrane preparations and the co-precipitated $\alpha2\delta1$ proteins were analyzed in Western blots (for further details see Methods and Figure 2). Gabap (Gabapentin); Pregab (Pregabalin).

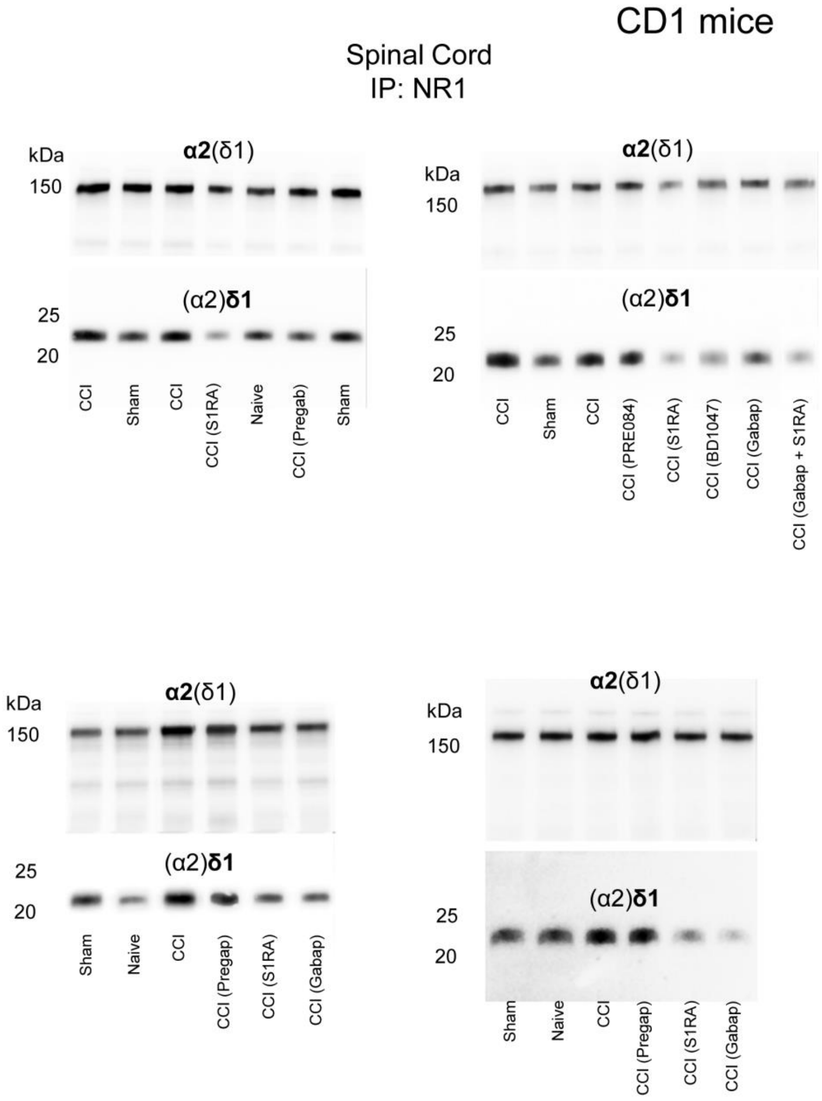

Figure S11. CCI model of neuropathic pain: the effect of S1RA, BD1047 and gabapentinoids on the α2δ1-NR1 association in spinal cord synaptosomal fraction (for details see Figure S10).
